# Genome-wide association study identifies 14 novel risk alleles associated with basal cell carcinoma

**DOI:** 10.1038/ncomms12510

**Published:** 2016-08-19

**Authors:** Harvind S. Chahal, Wenting Wu, Katherine J. Ransohoff, Lingyao Yang, Haley Hedlin, Manisha Desai, Yuan Lin, Hong-Ji Dai, Abrar A. Qureshi, Wen-Qing Li, Peter Kraft, David A. Hinds, Jean Y. Tang, Jiali Han, Kavita Y. Sarin

**Affiliations:** 1 Department of Dermatology, Stanford University School of Medicine, Stanford, California 94305, USA; 2 Department of Epidemiology, Richard M. Fairbanks School of Public Health, Melvin & Bren Simon Cancer Center, Indiana University, Indianapolis, Indiana 46202, USA; 3 Department of Medicine (Quantitative Sciences Unit), Stanford University School of Medicine, Stanford, California 94305, USA; 4 Department of Epidemiology and Biostatistics, Tianjin Medical University Cancer Hospital and Institute, National Clinical Research Center for Cancer, Tianjin & Key Laboratory of Cancer Prevention and Therapy, Tianjin, China; 5 Department of Dermatology, Warren Alpert Medical School, Brown University, Providence, Rhode Island 02903, USA; 6 Department of Epidemiology, School of Public Health, Brown University, Providence, Rhode Island 02903, USA; 7 Channing Division of Network Medicine, Department of Medicine, Brigham and Women's Hospital, Harvard Medical School, Boston, Massachusetts 02115, USA; 8 Department of Epidemiology, Harvard T.H. Chan School of Public Health, Boston, Massachusetts 02115, USA; 9 Department of Biostatistics, Harvard T.H. Chan School of Public Health, Boston, Massachusetts 02115, USA; 10 23andMe Inc., Mountain View, California 94040, USA

## Abstract

Basal cell carcinoma (BCC) is the most common cancer worldwide with an annual incidence of 2.8 million cases in the United States alone. Previous studies have demonstrated an association between 21 distinct genetic loci and BCC risk. Here, we report the results of a two-stage genome-wide association study of BCC, totalling 17,187 cases and 287,054 controls. We confirm 17 previously reported loci and identify 14 new susceptibility loci reaching genome-wide significance (*P*<5 × 10^−8^, logistic regression). These newly associated SNPs lie within predicted keratinocyte regulatory elements and in expression quantitative trait loci; furthermore, we identify candidate genes and non-coding RNAs involved in telomere maintenance, immune regulation and tumour progression, providing deeper insight into the pathogenesis of BCC.

## Abstract (web summary)

Basal cell carcinoma is a common skin lesion and the risk loci for this cancer are beginning to be understood. In this study, the authors conduct a two-stage genome-wide association study and confirm known risk loci and identify an additional 14 loci.

## Graphical abstract

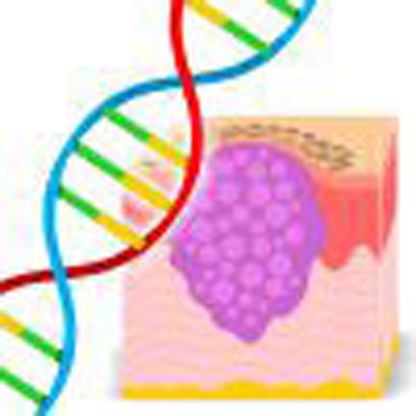

With 2.8 million new cases diagnosed annually in the United States^1^, basal cell carcinoma (BCC) is the most common cancer worldwide and contributes substantially to morbidity. BCC risk has been associated with ultraviolet (UV) exposure, fair skin, arsenic exposure, ionizing radiation, chronic immunosuppression, male gender and age^1^. Since 2008, six population-based genome-wide association studies (GWAS) of BCC have been reported identifying 16 regions of susceptibility^2^,^3^,^4^,^5^,^6^,^7^. Candidate gene studies have identified five additional pigmentation loci associated with BCC, including *IRF4*, *SLC45A2*, *RALY*, *TYR* and *OCA2*^7^. Here, we report the largest-to-date two-stage genome-wide association meta-analysis for BCC totalling 17,187 cases and 287,054 controls. The results of this study confirm 12 of 16 loci from prior GWAS, along with 5 of 5 loci from previous candidate gene studies, and identify 14 novel susceptibility loci for BCC.

## Results

Stage 1 consisted of 12,945 self-reported BCC cases and 274,252 controls of European ancestry from 23andMe research participants (Supplementary Table 1). Validation of self-reported surveys with adjudicated medical records revealed a sensitivity and specificity of 93% and 99%, respectively (Supplementary Table 2), indicating a misclassification rate of less than 10%. Simulation analysis demonstrated that this misclassification rate only modestly reduced the power to detect associations in this study (Supplementary Fig. 1). Stage 2 consisted of an independent GWAS cohort of 4,242 BCC cases and 12,802 controls of European ancestry from the Nurses' Health Study and Health Professionals Follow-Up Study (Supplementary Table 1). Subsequently, meta-analysis of stages 1 and 2 was performed, encompassing 17,187 cases and 287,054 controls (Supplementary Table 1). Further information on methodology and imputation quality control is presented in the Online Methods, Supplementary Tables 3-5, and Supplementary Figs 2–7.

### Stage 1 analysis

**Figure 1:**
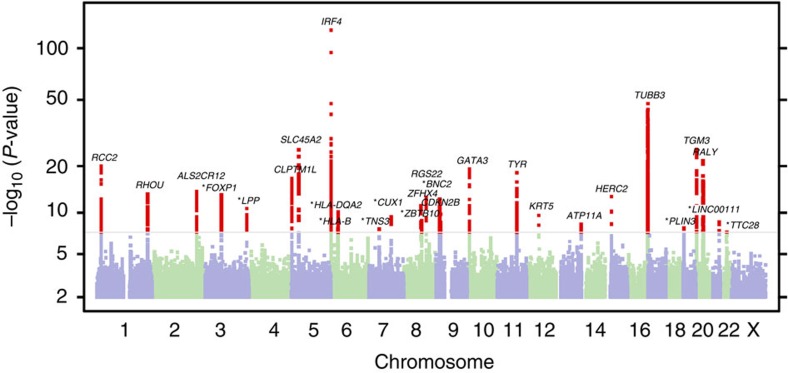
Manhattan plot of stage 1 GWAS analysis of BCC from 23andMe data set. Total stage 1 GWAS analysis included 12,945 cases and 274,252 controls. Loci with smallest *P*<10^−6^ (28 total, logistic regression) are labelled with the name of the nearest gene. Positions with *P*<5 × 10^−8^ (genome-wide significance) are shown in red. In stage 1, ten novel BCC susceptibility loci reached genome-wide significance after adjusting for genomic control, all of which are labelled in the figure with asterisks: from left to right, **3p13** (*FOXP1*), **3q28** (*LPP*), **6p21.32** (*HLA-DQA2*), **6p21.33** (*HLA-B*), **7p12.3** *(TNS3*), **7q22.1** (*CUX1*), **8q21.13** (*ZBTB10*), **9p22.2** (near *BNC2*), **19p13.3** (*PLIN3*), **21q22.3** (*LINC00111*). Four additional novel susceptibility loci—**6p21.3** (*NEU1*), **10q24.3** (*OBFC1*), **6q27** (*MIR3939*), **6p22.3**(*CASC15*)—were genome-wide significant in the overall meta-analysis (Table 2) and thus are not labelled in the figure.

Twenty-eight index single nucleotide polymorphisms (SNPs) were associated with BCC at the genome-wide significance level (*P*<5.0 × 10^−8^, logistic regression) in stage 1 and are depicted in the Manhattan plot (Fig. 1). Subset analysis revealed relatively consistent effect sizes across age and gender for these 28 SNPs (Supplementary Tables 6–7, Supplementary Fig. 8). Interestingly, slightly larger effect sizes tended to occur in younger cases, suggesting that other risk factors may play an increasing role with age. As 10% of the BCC cases in our stage 1 cohort subsequently developed melanoma, we also investigated whether the co-occurrence of melanoma contributed to the observed associations with BCC risk. We therefore computed association tests for the stage 1 index SNPs in BCC cases with and without melanoma. All 28 SNPs displayed consistent effect sizes across the two groups (Table 1, Supplementary Fig. 9), indicating that they are independently associated with BCC susceptibility.

### Stage 2 and combined meta-analysis

Twenty of the 28 index SNPs were replicated in the stage 2 analysis (*P*<0.05, logistic regression). While some loci did not reach statistical significance in stage 2, their 95% confidence intervals (for odds ratios) overlapped with the corresponding stage 1 confidence intervals. Meta-analysis of stages 1 and 2 identified a total of 31 loci reaching genome-wide significance (*P*<5 × 10^−8^, logistic regression) for association with BCC. Among these 31 loci, 17 are previously reported (Supplementary Tables 8–12). The remaining 14 are novel BCC susceptibility loci (Table 2, Supplementary Table 13). Forest plots and regional association plots for these 14 SNPs are provided in Supplementary Figs 10–12. Of these 14 novel risk variants, 10 reached genome-wide significance in stage 1 and 4 reached genome-wide significance in the combined meta-analysis. As many pigmentation loci have been associated with BCC susceptibility, we adjusted for pigmentation phenotype in our stage 2 cohort and did not observe a significant difference between adjusted and unadjusted results for the 14 novel risk variants (Supplementary Table 14).

### Heritability of BCC and gene expression analysis

**Figure 2:**
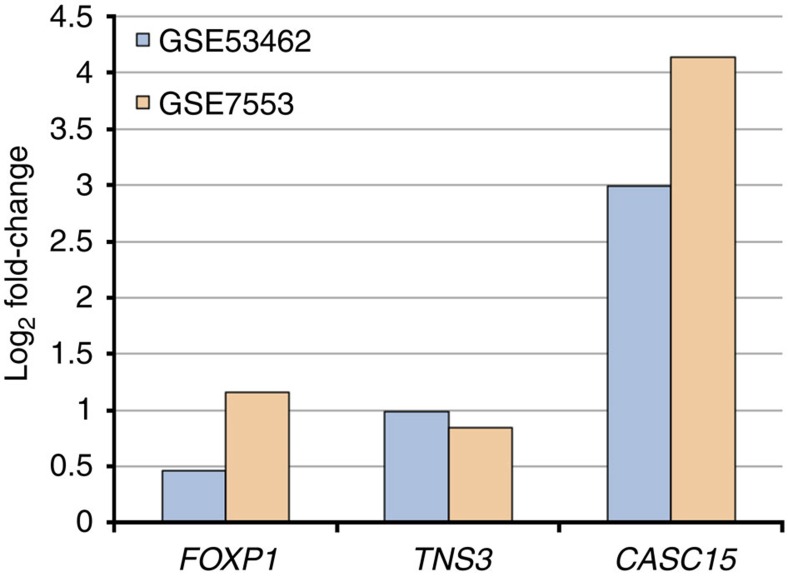
Gene expression analysis for novel BCC susceptibility loci. Processed microarray expression data were obtained from Gene Expression Omnibus (GSE53462, blue, and GSE7553, orange). Transcript levels in BCC samples were compared to levels in normal skin controls via Geo2R. Three genes—*FOXP1*, *TNS3* and *CASC15*—were significantly upregulated in BCC relative to normal skin (*P*<0.05, linear models for microarray analysis) in both data sets.

To measure the proportion of BCC heritability that can be attributed to these SNPs, we calculated the familial relative risk for BCC as outlined by the Cancer Oncological Gene-Environment Study. Overall, 10.98% of familial relative risk for BCC is explained by the 31 genome-wide significant loci; of this percentage, the 14 novel susceptibility loci account for 2.62%. To further explore the role of these 14 loci in BCC pathogenesis, we evaluated expression levels of nearby genes in BCC tissue using two data sets from the Gene Expression Omnibus (GEO) (GSE53462 and GSE7553). Three loci demonstrated significant differential gene expression in BCC relative to normal skin: 3p13 *FOXP1*, 7p12.3 *TNS3* and 6p22.3 *CASC15* (Fig. 2, *P*<0.05, linear models for microarray analysis).

### Gene-environment interaction analysis

Ultraviolet exposure and pigmentation phenotypes have been associated with BCC risk and may interact with genetic variants to confer BCC susceptibility. To further explore the influence of such factors on our study, we tested for interactions between all 31 significant loci and UV exposure, hair colour, number of sunburns and tanning ability (Supplementary Table 15). This analysis revealed only one significant interaction, between rs191177147 (*LPP*) and hair colour. Additional testing in three separate hair colour groups demonstrated that rs191177147 significantly interacted with the light brown and dark brown/black hair groups (Table 3, *P*=2.9 × 10^−6^, logistic regression).

## Discussion

The 14 novel susceptibility loci cluster into five functional categories: telomere biology, immune regulation, tumour progression, non-coding RNA and pigmentation.

Variants within the *TERT* locus have previously been associated with BCC susceptibility, thus implicating telomere regulation in BCC development^8^. Here, we identify a novel BCC susceptibility locus, *OBFC1*, that is also involved in telomere maintenance. rs7907606 at 10q24.3 (*P*=4.7 × 10^−9^, odds ratio (OR)=1.10, logistic regression) lies 3 kb upstream of the *OBFC1* gene, a member of the heterotrimer CST complex^8^. This complex restricts telomere extension by binding telomeric DNA and disrupting the ability of other proteins to recruit telomerase^9^. Variants in the *OBFC1* locus have been associated with mean leukocyte telomere length^8^,^10^. rs7907606 is in linkage disequilibrium (LD) with four of these variants (Supplementary Table 16), including rs4387287 (*r*^2^=0.98, *D*′=−1.00), which lies within the promoter of *OBFC1* and is an eQTL for this gene in sun-exposed skin (*P*=8.3 × 10^−6^, GTEx V6 analysis)^11^. Though widely studied, the connection between telomere length and cancer is context dependent, as both long and short telomeres are linked to malignancy^12^. This discrepancy is readily apparent in the context of skin cancer, where longer telomeres are associated with melanoma and shorter telomeres are associated with BCC^12^. Our results further implicate telomere homeostasis in BCC pathogenesis.

We also identified six novel susceptibility loci associated with immune regulation. Two SNPs, rs1050529 and rs9275642, are in human leukocyte antigen (HLA) regions. rs1050529 at 6p21.33 (*P*=2.6 × 10^−9^, OR=0.90, logistic regression) lies within an exon of *HLA-B*, and leads to a non-conservative amino acid substitution (A65T). rs1050529 has predicted enhancer and promoter activity in keratinocytes and is an eQTL in six tissues. Variants in *HLA-B* have been associated with a range of autoimmune conditions, including vitiligo and psoriasis^13^,^14^. *HLA-B* encodes the heavy chain component of major histocompatibility complex (MHC) class I molecules, which present endogenously synthesized peptides to cytotoxic T cells. Downregulation of MHC class I molecules is a feature of many types of cancers, which is thought to enable tumour cells to evade recognition and destruction by T cells^15^. An analysis of 91 human melanoma cell lines revealed decreased expression of class I molecules in 67% of the cell lines, with *HLA-B* being the most common^16^. The second HLA SNP reaching genome-wide significance was rs9275642 at 6p21.32 (*P*=2.4 × 10^−12^, OR=0.89, logistic regression), which is located 24 kb upstream of *HLA-DQA2*. This SNP is in tight LD with rs9275640 (*r*^2^=0.89, D′=0.99), which is an eQTL for this gene in sun-exposed skin (*P*=3.6 × 10^−6^, GTEx V6 analysis)^11^. *HLA-DQA2* variants are also associated with autoimmune diseases, including type 1 diabetes, rheumatoid arthritis and alopecia areata^14^. These findings represent the first genome-wide significant association between MHC genes and BCC risk.

The third immune-related SNP, rs191177147 at 3q28 (*P=*9.8 × 10^−17^, OR=1.11, logistic regression), resides within an intron of *LPP* and is in LD with rs1464510 (*r*^2^=0.54) and rs9860547 (*r*^2^=0.68, Supplementary Table 17); the former is associated with autoimmune diseases such as celiac disease, rheumatoid arthritis, juvenile idiopathic arthritis and vitiligo^17^,^18^,^19^, while the latter is associated with allergy^20^. *LPP* encodes an intracellular protein that shuttles between the cell membrane and the nucleus, where it interacts with transcription factors to modulate gene expression^21^. *LPP* overexpression has been reported in squamous cell lung carcinomas and primary sarcomas^21^. We also found an association between rs191177147 and hair colour, suggesting that this SNP may also contribute to BCC risk by altering pigmentation phenotypes.

Another SNP with potential significance in immune regulation is rs10425559 at 19p13.3 (*P=*2.9 × 10^−9^, OR=0.93, logistic regression), which is intergenic and flanked by *TICAM1* and *PLIN3*. It is linked to rs7255265 (*r*^2^=0.68, *D*′=0.9, Supplementary Table 18), which is located in an exon of *TICAM1* and has predicted enhancer activity in keratinocytes. TICAM1 is an intracellular toll-like receptor(TLR) adaptor molecule involved in innate immunity; moreover, it acts as a pro-apoptotic tumour suppressor by mediating the interaction between TLR-3 and caspase-8 in some malignancies, including melanoma^22^. *PLIN3* encodes a cytosolic protein that binds to the GTPase RAB9, a member of the RAS oncogene family^23^. Overexpression of *PLIN3* has been linked to cervical carcinoma^24^.

The fifth immune-related SNP, rs9267650 at 6p21.3 (*P=*1.1 × 10^−8^, OR=1.17, logistic regression), lies 0.5 kb downstream of *NEU1*, which encodes a lysosomal enzyme implicated in many diverse processes, including activation of TLRs^25^, wound healing^26^,^27^ and suppression of ovarian carcinoma^28^. Finally, rs11993814 at 8q21.13 (*P*=1.1 × 10^−12^, OR=0.91, logistic regression), located 9 kb upstream of *ZBTB10*, is an eQTL in two tissues and is in LD with rs6998967 (*r*^2^=0.6, *D*′=1.0), associated with late-onset myasthenia gravis^29^. *ZBTB10* encodes a zinc finger transcription factor involved in regulating the expression of Interleukin-10 through suppression of Sp1^29^,^30^,^31^. Abnormal interaction between ZBTB10 and Sp1 is seen in several different cancer cell lines, with ZBTB10 consistently exhibiting tumour-suppressing activity^32^. All together, these findings implicate a number of immune regulatory loci in BCC susceptibility.

Four of our novel susceptibility loci are associated with tumour progression. rs2116709 at 3p13 (*P=*5.7 × 10^−16^, OR=0.91, logistic regression) resides within an intron of *FOXP1*, a transcription factor that, in addition to regulating organ development, acts as a tumour suppressor in some cancers (for example, prostate^33^) and as an oncoprotein in others (for example, oesophagus^34^). It is overexpressed in oesophageal cancer and many types of lymphomas, including cutaneous B-cell lymphomas^34^,^35^. Similarly, we found that *FOXP1* is significantly overexpressed in BCC as compared with normal skin controls. Our findings suggest a role for *FOXP1* in BCC development.

rs73183643 at 7q22.1 (*P=*1.3 × 10^−13^, OR=0.91, logistic regression) is located 40 kb upstream of *CUX1*, which encodes a homeodomain-containing transcription factor involved in cell proliferation and differentiation. Both *in vitro* and *in vivo* studies have shown that *CUX1* promotes tumorigenesis in a range of neoplasms, including melanoma and pancreatic cancer, by increasing cell motility and inhibiting apoptosis^36^,^37^.

Another tumorigenesis-related SNP, rs7776701 at 7p12.3 (*P=*2.0 × 10^−8^, OR=0.94, logistic regression), lies within an intron of *TNS3* and has enhancer activity in 14 tissues, including keratinocytes. This SNP is in LD with rs56232506 (*r*^2^=0.76, *D*′=0.99), also intronic to *TNS3* and associated with prostate cancer^38^. Tensin-3, the cytosolic protein product of *TNS3*, connects transmembrane proteins to cytoskeletal elements and influences cell migration^39^. Studies of human metastatic melanoma, non-small cell lung cancer and breast cancer cell lines demonstrate that reduced expression of *TNS3* corresponds to dramatic inhibition of cell proliferation and migration, suggesting that *TNS3* is an oncogene^39^. This idea is consistent with our expression analysis, in which *TNS3* was significantly upregulated in BCC.

The fourth SNP in this category, rs4710154 at 6q27 (*P*=1.1 × 10^−8^, OR=1.08, logistic regression), lies 17 kb downstream of *MIR3939*, which codes for microRNA 3939. Despite its proximity to *MIR3939*, rs4710154 is an eQTL for *RNASET2* in 11 tissues, including sun-exposed skin (*P*=4.7 × 10^−19^, GTEx V6 analysis) and non-sun-exposed skin (*P*=1.1 × 10^−7^, GTEx V6 analysis)^11^. This SNP is linked to rs9355610 (*r*^2^=0.89, *D*′=0.99, Supplementary Table 19), associated with Grave's disease and Hashimoto's thyroiditis^40^; rs9355610 is also an eQTL for *RNASET2* in sun-exposed (*P*=1.0 × 10^−1^, GTEx V6 analysis) and non-sun-exposed skin (*P*=1.6 × 10^−8^, GTEx V6 analysis)^11^. *RNASET2* encodes ribonuclease T2, an evolutionarily conserved, ubiquitous RNase that inhibits cell proliferation (via stimulation of immune cells) and mediates cellular stress responses^41^. Accordingly, this enzyme has tumour suppressor activity in many cancer lines, including melanoma^42^, and has demonstrated pro-apoptotic activity in keratinocytes^43^.

Interestingly, two of the 14 novel susceptibility variants reside near or within long non-coding RNA genes. rs2776353 at 21q22.3 (*P=*2.0 × 10^−14^, OR=0.91, logistic regression) is located 10 kb upstream of *LINC00111*, while rs2294214 (*P=*3.1 × 10^−8^, OR=1.07, logistic regression) lies within an intron of *CASC15* (also known as *LINC00340*); both SNPs have predicted enhancer and promoter activity in keratinocytes. A recent study compared the expression of long non-coding RNAs in BCC samples to that of normal skin controls and found that *CASC15* was overexpressed in BCC^44^. Overexpression of *CASC15* has also been implicated in melanoma progression and metastasis^45^. Our analysis of independent BCC expression data confirmed significant upregulation of *CASC15* in BCC, further implicating this gene in BCC development.

In addition to confirming the previously reported association of five pigmentation loci with BCC, we identified a novel susceptibility locus—rs10810657 at 9p22.2 (*P*=1.5 × 10^−17^, OR=0.90, logistic regression)—that may also influence pigmentation. rs10810657, located 14 kb upstream of *BNC2*, reached genome-wide significance in the meta-analysis and is an eQTL for *BNC2* in blood (Table 2). This SNP is in LD with rs12350739 (*r*^2^=0.87, *D*′=0.99, Supplementary Table 20), associated with human skin pigmentation via regulation of *BNC2* transcription in melanocytes, and rs62543565 (*r*^2^=0.7, *D*′=0.87), associated with the age-related development of facial pigmented spots^46^,^47^. rs10810657 is also linked to rs2153271 (*r*^2^=0.9, *D*′=0.99), which is intronic to *BNC2* and associated with freckling^48^. *BNC2* codes for basonuclin 2, a protein thought to function both as an mRNA-processing enzyme and a transcription factor^46^. It is expressed in melanocytes and, to a lesser extent, keratinocytes, with higher expression levels corresponding to darker skin pigmentation^46^.

In summary, this two-stage meta-analysis represents the largest GWAS for BCC and identified 14 novel susceptibility loci with roles in telomere maintenance, immune regulation and tumour progression. Further investigation of these loci will improve our understanding of BCC pathogenesis and improve our ability to prevent these common tumours.

## Methods

### Stage 1 study design and population

23andMe Inc. (Mountain View, CA), a genetics company, provided free access to anonymized genetic and phenotypic information for stage 1 of this GWAS. All information came from 23andMe research participants who provided informed consent to participate in research, in accord with 23andMe's human subjects protocol (reviewed and approved by Ethical and Independent Review Services, an AAHRPP accredited IRB). 23andMe gathers genetic information by genotyping sample material provided by its research participants; phenotypic information is collected via research participant responses to online surveys. Inclusion and exclusion criteria are discussed below. Sample sizes for cases and controls were not predetermined as the intention was to maximize the number of subjects in both groups; hence, all subjects passing inclusion and exclusion criteria were included, which resulted in 12,945 BCC cases and 274,252 controls.

### Stage 1 genome-wide association analysis

Association analysis for stage 1 was performed using logistic regression, assuming an additive model for allelic effects. The analysis was adjusted for age, sex and population stratification (using the first five principal components), generating the following model:


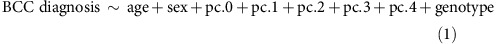


The association test *P*-value was computed using a likelihood ratio test. Results for the X chromosome were computed similarly, with male genotypes coded as if they were homozygous diploid for the observed allele. Additionally, test statistics were adjusted for genomic control to correct for residual population stratification persisting after principal component analysis; the genomic control inflation factor was 1.085 (computed from the median *P*-value for results that passed quality control).

Genome-wide association analysis generated a set of index SNPs. The index SNPs show information for the most-associated SNP in each associated region. We define regions of interest by identifying SNPs with *P*<10^−5^, then grouping these into intervals separated by gaps of at least 250 kb, and choosing the SNP with smallest *P* within each interval.

### Stage 1 genotyping and quality control

Samples were genotyped on one of four genotyping platforms. The V1 and V2 platforms were variants of the Illumina HumanHap550+ BeadChip, including about 25,000 custom SNPs selected by 23andMe, with a total of about 560,000 SNPs. The V3 platform was based on the Illumina OmniExpress+ BeadChip, with custom content to improve the overlap with our V2 array, with a total of about 950,000 SNPs. The V4 platform in current use is a fully custom array, including a lower redundancy subset of V2 and V3 SNPs with additional coverage of lower-frequency coding variation, and about 570,000 SNPs. Samples that failed to reach 98.5% call rate were re-analysed. Individuals whose analyses failed repeatedly were re-contacted by 23andMe to provide additional samples, as is done for all 23andMe research participants.

Individuals were only included if they had >97% European ancestry, as determined through an analysis of local ancestry^49^. Briefly, this analysis first partitions phased genomic data into short windows of about 100 SNPs. Within each window, a support vector machine is used to classify individual haplotypes into one of 31 reference populations. The support vector machine classifications are then fed into a hidden Markov model (HMM) that accounts for switch errors and incorrect assignments, and gives probabilities for each reference population in each window. Finally, simulated admixed individuals are used to recalibrate the HMM probabilities so that the reported assignments are consistent with the simulated admixture proportions. The reference population data are derived from public data sets (the Human Genome Diversity Project, HapMap and 1000 Genomes), as well as 23andMe research participants who have reported having four grandparents from the same country.

A maximal set of unrelated individuals was chosen for each analysis using a segmental identity-by-descent (IBD) estimation algorithm^50^. Individuals were defined as related if they shared more than 700 cM IBD, including regions where the two individuals share either one or both genomic segments identical-by-descent. This level of relatedness (roughly 20% of the genome) corresponds approximately to the minimal expected sharing between first cousins in an outbred population.

Participant genotype data were imputed against the March 2012 ‘v3' release of 1000 Genomes reference haplotypes^51^. Data for each genotyping platform were phased and imputed separately. First, Beagle^52^ (version 3.3.1) was used to phase batches of 8000–9000 individuals across chromosomal segments of no more than 10,000 genotyped SNPs, with overlaps of 200 SNPs. SNPs with Hardy-Weinberg equilibrium *P*<10^−20^, call rate<95%, or with large allele frequency discrepancies compared to European 1000 Genomes reference data were excluded. Frequency discrepancies were identified by computing a 2 × 2 table of allele counts for European 1000 Genomes samples and 2000 randomly sampled 23andMe research participants with European ancestry, and identifying SNPs with a chi-squared *P*<10^−15^. Each phased segment was imputed against all-ethnicity 1000 Genomes haplotypes (excluding monomorphic and singleton sites) using Minimac2^53^, using 5 rounds and 200 states for parameter estimation.

For the non-pseudoautosomal region of the X chromosome, males and females were phased together in segments, treating the males as already phased; the pseudoautosomal regions were phased separately. Males and females were then imputed together using minimac, as with the autosomes, treating males as homozygous pseudo-diploids for the non-pseudoautosomal region.

For quality control of genotyped GWAS results, SNPs that were only genotyped on the ‘V1' platform were flagged due to small sample size, and SNPs on chrM or chrY because many of these are not currently called reliably. Using trio data, SNPs that failed a test for parent-offspring transmission were also flagged; specifically, the child's allele count was regressed against the mean parental allele count, and SNPs with fitted *β*<0.6 and *P*<10^−20^ for a test of *β*<1 were flagged. SNPs with a Hardy-Weinberg *P*<10^−20^ in Europeans, or a call rate of <90%, were also flagged. Genotyped SNPs were also tested for genotype date effects, and SNPs with *P*<10^−50^ by analysis of variance of SNP genotypes against a factor dividing genotyping date into 20 roughly equal-sized buckets were flagged.

For imputed GWAS results, SNPs with avg.rsq<0.5 or min.rsq<0.3 in any imputation batch were flagged, as well as SNPs that had strong evidence of an imputation batch effect. The batch effect test was an F test from an analysis of variance of the SNP dosages against a factor representing imputation batch; results with *P*<10^−50^ were flagged. Prior to GWAS, the largest subset of the data passing these criteria was identified for each SNP, based on their original genotyping platform—either v2+v3+v4, v3+v4, v3, or v4 only—and association test results were computed for whatever was the largest passing set. As a result, there were no imputed results for SNPs that failed these filters.

When choosing between imputed and genotyped GWAS results, if either the imputed test passed quality control, or a genotyped test was unavailable, the imputed result was reported; otherwise, the genotyped result was reported. For tests using imputed data, imputed dosages were used rather than best-guess genotypes.

Across all results, logistic regression results that did not converge due to complete separation, identified by abs (effect)>10 or stderr>10 on the log odds scale, were flagged. Linear regression results for SNPs with MAF<0.1% were also flagged, since tests of low frequency variants can be sensitive to violations of the regression assumption of normally distributed residuals^20^,^54^,^55^.

### Stage 1 associations using nearest genotyped SNP

To assess the effect of imputation, we analysed the association between the nearest genotyped SNP at each locus and BCC, and then compared this association to that from the original imputed SNP. The genotyped results are consistent with the imputed results albeit slightly less significant.

### Stage 1 subset analyses

Subset analysis by age and gender was performed for the genome-wide significant index SNPs in stage 1. For age-based analysis, the stage 1 cohort was divided into four age intervals with similar effective sample sizes based on case and control sample counts. Association test results were then computed within each of these age intervals for the 28 SNPs. The interaction between genotype effect and age interval was also calculated. For all these association tests, we used the same covariates used in stage 1: age, sex and five principal components. Thus, association tests within a specific age interval were still adjusted for age as a continuous covariate. For gender analysis, we compared effect sizes estimated in men versus effect sizes estimated in women for the 28 SNPs. We also performed logistic regression separately in the male and female subsets, and calculated *P*-values from a likelihood ratio test for adding a gender by genotype interaction to the full logistic regression models. For melanoma subset analysis, association tests for BCC were computed separately in melanoma controls and melanoma cases.

### Stage 1 phenotype categorization

23andMe identified BCC cases by using research participants' self-reported answers to online questionnaires. Subjects who answered ‘Yes' and/or selected BCC from a dropdown menu in response to at least one of the following questions were defined as cases: ‘Have you ever been diagnosed by a doctor with basal cell carcinoma?', ‘What type of skin cancer did you have? Please check all that apply.', ‘What type of skin cancer or cancers have you been diagnosed with? Please check all that apply.' ‘Have you ever been diagnosed with basal cell carcinoma?' ‘Have you ever been diagnosed or treated for any of the following conditions?' Controls were defined as subjects who answered ‘No' and did not select BCC from any relevant dropdown menus. In addition, subjects who answered ‘No' to at least one of the following questions (and ‘Yes' to none) were defined as controls: ‘Have you ever been diagnosed with cancer, including skin cancer or cancerous moles?', ‘Has a doctor ever told you that you have a type of cancer?', ‘Have you ever been diagnosed or treated with any of the following conditions?' Among the samples with imputed genotypes, 23andMe has 12,945 BCC cases and 274,252 controls.

### Sensitivity and specificity of stage 1 self-reported data

To assess the validity of self-reported phenotypic data in stage 1, 23andMe surveys (pertaining to skin cancer history and pigmentation) were randomly administered to patients seen in Stanford outpatient clinics. The survey answers were then compared to medical records to assess for accuracy with respect to BCC diagnosis to determine the sensitivity and specificity of the survey responses. *P*-values were determined using chi-square analysis. This sub-study was approved by the Stanford University Institutional Review Board with a waiver of documentation of informed consent.

### Stage 2 study design and population

The Nurses' Health Study was established in 1976, when 121 700 female registered nurses between the ages of 30 and 55 years residing in 11 larger US states completed and returned an initial self-administered questionnaire on their medical histories and baseline health-related exposures. Biennial questionnaires with collection of exposure information on risk factors have been collected prospectively. Every 2 years, along with exposures, outcome data with appropriate follow-up of reported disease events are collected. Overall, follow-up has been high; after more than 20 years, ∼90% of participants continue to complete questionnaires. From May 1989 through September 1990, we collected blood samples from 32,826 participants in the NHS. Information on BCC development was first collected in the 1984 questionnaire.

The Health Professionals Follow-up Study (HPFS) was established in 1986 when 51,529 men from all 50 US states in health professions (dentists, pharmacists, optometrists, osteopath physicians, podiatrists and veterinarians) aged 40–75 years answered a detailed mailed questionnaire. The average follow-up rate for this cohort over 10 years is >90%. On each biennial questionnaire, we obtained disease- and health-related information. Between 1993 and 1994, 18,159 study participants provided blood samples by overnight courier. Information on BCC development was first collected in the 1986 questionnaire.

The protocol for this study was approved by the Institutional Review Board at Brigham and Women's Hospital and the Harvard School of Public Health. All of the participants provided informed consent. As in stage 1, all subjects passing inclusion and exclusion criteria were included, resulting in 4,242 BCC cases and 12,802 controls; sample sizes were not predetermined.

### Stage 2 genotyping and quality control

There were 18 GWAS data sets from the NHS and HPFS as nested case-control studies with cleaned genotype data available. We combined these data sets into three complied data sets based on their genotype platform type: Affymetrix, Illumina HumanHap series or Illumina Omni Express. The Affymetrix data set was comprised of data on the Affy 6.0 platform (NHS-type 2 diabetes, NHS-coronary heart disease, HPFS-type 2 diabetes, HPFS-coronary heart disease). The Illumia HumanHap data set was comprised of several platforms: Illumina 550K (NHS-breast cancer, NHS-Pancreas cancer, HPFS-pancreas cancer), Illumina 610Q (NHS-kidney stone, HPFS-kidney stone, HPFS-prostate cancer) and Illumina 660 (NHS-glaucoma, HPFS-glaucoma). The Illumina Omni Express data set contained only studies genotyped on the Omni Express platform (NHS-endometrial cancer, NHS-colon cancer, NHS-mammographic density, NHS-gout, HPFS-colon, HPFS-gout).

We combined the individual data sets that were genotyped on the same platform, removing any SNPs that were not in all studies and with a missing call rate>5%, and flipping strands where appropriate to create a final compiled data set. This resulted in 668,283 SNPs in the Affymetrix data set, 459,999 SNPs in the Illumina HumanHap data set and 565,810 SNPs in the Illumina Omni Express data set. Analyses were restricted to subjects with self-reported European ancestry. Genetic principal components were calculated using sets of independent SNPs (12,000–33,000 SNPs depending on platform). Subjects who did not cluster with other self-identified Europeans based on the top five principal components were also excluded.

We then ran a pairwise IBD analysis for each combined data set to detect duplicate and related individuals based on resulting *Z* scores. If 0⩽*Z*0⩽0.1 and 0⩽*Z*1⩽0.1 and 0.9 ⩽*Z*2⩽1.1 then a pair was flagged as being identical twins or duplicates. Pairs were considered full siblings if 0.17⩽*Z*0⩽0.33 and 0.4⩽*Z*1⩽0.6 and 0.17⩽*Z*2⩽0.33. Half siblings or avunculars were defined as having 0.4⩽*Z*1⩽0.6 and 0⩽*Z*2⩽0.1. Some of the duplicates flagged in this step were expected, having been genotyped in multiple data sets and hence having the same cohort IDs. In this case, one of each pair was randomly chosen for removal from the data set. Instances where pairs were flagged as unexpected duplicates with the different cohort IDs, but pairwise genotype concordance rate>0.999, resulted in removal of both individuals from the pair. Related individuals (full sibs, half sibs/avunculars) were not removed from the final data sets. In the Affymetrix data set, 167 individuals were removed because they were duplicates or were flagged for removal from secondary genotype data cleaning, leaving a total of 8065 individuals. Of the 6894 individuals originally in the Illumina data set, 107 were removed because they were duplicates or flagged for removal in the genotyping step, leaving 6787 IDs. In addition, eight pairs of individuals were flagged as related. In the Omni express data set, there were 5956 individuals at the start, with 39 IDs to remove leaving 5917 IDs and 5 pairs of related IDs.

After removing duplicate IDs and flagging related pairs of IDs, we used eigenstrat to run PCA analysis on each compiled data set, removing one member from each flagged pair of related individuals. For Affymetrix and Illumina HumanHap, we used approximately 12,000 SNPs that were filtered to ensure low pairwise LD^56^. For the OmniExpress data set we used approximately 33,000 SNPs that were similarly filtered. We plotted the top eigenvectors using R and examined the plots for outliers.

Finally as a quality control check, we ran logistic regression analyses using each individual study's controls as ‘cases' and the rest of the studies controls as ‘controls'. For example, in the Illumina Omni Express data set, we ran regressions of NHS-gout controls considered as ‘cases' versus the HPFS-gout, NHS-endometrial cancer, NHS-colon cancer, NHS-mammographic density and HPFS-colon cancer. We then ran regressions with each of the other study controls as ‘cases' versus all of the rest of the controls. We looked for *P* values of genome-wide significance (*P*<10^−8^) and examined QQ plots to determine if any SNPs were flagged as significant where no SNPs should have been significant. In the Affymetrix data set 100 SNPs were flagged and removed. In the Illumina HumanHap data set, eight SNPs had *P*<10^−8^ in any of the QC regressions and were removed. No SNPs in the Illumina Omni Express data set had *P*-values<10^−8^ hence no additional SNPs needed to be removed. After the data sets were combined and appropriate SNP and ID filters applied, the complied data sets were imputed.

Using combined GWAS genotypes on each genotyping platform and the 1000 Genomes Project ALL Phase I Integrated Release Version 3 Haplotypes excluding monomorphic and singleton sites (2010-11 data freeze, 2012-03-14 haplotypes) as reference panel, we imputed the genotypes of markers in the 1000 Genomes Project for 8065 samples in Affymetrix data set, 6787 samples in Illumina HumanHap data set and 5917 samples in Illumina Omni Express data set.

SNP genotypes were imputed in three steps. First, genotypes on each chromosome were split into chunks to facilitate windowed imputation in parallel using ChunkChromosome (v.2011-08-05) (http://genome.sph.umich.edu/wiki/ChunkChromosome). Then each chunk of chromosome was phased using MACH (v.1.0.18.c) (http://www.sph.umich.edu/csg/abecasis/MaCH/index.html). In the final step, Minimac (v.2012-08-15) (http://genome.sph.umich.edu/wiki/Minimac) was used to impute the phased genotypes to approximately 31 million markers in the 1000 Genomes Project^51^.

### Sensitivity analysis in stage 2

Sensitivity analysis for five SNPs—*IRF4* rs12203592, *KRT5* rs11170164, *PLIN3* rs10425559, *NEU1* rs9267650 and *EXOC2* rs12210050—using high imputation quality subsets was performed. Concordance for two SNPs (rs12916300 and rs35407) was compared between stage 2 imputed data and directly genotyped data among the overlapping samples of 251 controls and 280 cases. Direct genotyping was also performed for rs9275642 and rs1050529 in a subset of BCC cases and controls within NHS. The total sample size is 1204 with 661 cases and 543 controls.

### Stage 2 phenotype categorization

Participants in both NHS and HPFS cohorts reported new BCC diagnosis biennially. Eligible cases in the NHS and HPFS consisted of participants with self-reported BCC any time after baseline up to the 2012 follow-up cycle for both cohorts. Samples free of BCC were controls in this study. In the three compiled data sets, samples without information on BCC diagnosis were excluded. Furthermore, all BCC cases with melanoma were also excluded. Among the samples with imputed genotypes, we have 1777 BCC cases and 5411 controls in Affymetrix data set, 1268 BCC cases and 3685 controls in Illumina HumanHap data set and 1197 BCC cases and 3706 controls in Illumina Omni Express data set totalling 4242 BCC cases and 12,802 controls.

### Validity of stage 2 self-reported data

The identification of BCC cases in stage 2 was based on self-report without pathological confirmation. Because the participants in the cohorts were nurses and other health professionals, the validity of their reports was expected to be high and has been proven in validation studies: >90% confirmed by histopathology records^57^,^58^,^59^. In addition, previous studies of BCC in the NHS using self-reported cases identified both constitutional and sun-exposure risk factors as expected, such as lighter pigmentation, less childhood and adolescent tanning tendency, higher tendency to sunburn, and tanning salon attendance^58^,^60^. Moreover, in our previous study of BCC using the same cohorts as this study (NHS and HPFS), we confirmed the *MC1R* gene (a well-established pigmentation gene) as the most promising locus of BCC risk^4^. In addition, we identified many other pigmentation SNPs (for example, rs12203592 at *IRF4*, rs1408799 at *TYRP1* and rs12913832 at *HERC2*) in our data set with significant associations with BCC^4^. These genetic and non-genetic data together suggest that the bias due to self-report of BCC is likely to be minimal in our study.

### Stage 2 genome-wide association analysis

We used ProbABEL software to test the GWAS association between minor allele counts and BCC risk using imputed dosage data. We performed logistic regression analysis under an additive model with adjustment for age, sex, BCC history and the first five principal components, generating the following model:


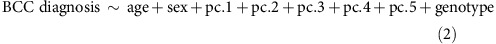


These principal components were calculated for all individuals on the basis of approximately 10,000 unlinked markers using the EIGENSTRAT software^61^. Associations in each component GWAS set (Affymetrix, Illumina HumanHap series and Illumina Omni Express) were combined in an inverse-variance-weighted meta-analysis using the METAL software.

### Phenotype and environmental factors

The amount of UV exposure based on residence location in 1986 was coded as a continuous variable. Information on pigmentation traits was collected from prospective questionnaires in both the NHS and HPFS using similar wording. We regressed ordinal coding for natural hair colour (1—red, 2—blonde, 3—light brown, 4—dark brown or black), tanning ability during adolescence (1—tans without burning (none/some redness only), 2—burns then tans, 3—burns/peels (painful burn with blisters)) and number of blistering sunburns (1=never, 2=1-2 times, 3=3–5 times, 4=6 times and above).

### Statistical models for pigmentation and UV exposure

To clarify the influence of pigmentation traits on our associations, we additionally adjusted for these traits (hair colour, tanning ability during adolescence and number of blistering sunburns) in individual studies with ProbABEL. Associations in each component GWAS set (Affymetrix, Illumina HumanHap series and Illumina Omni Express) were combined in the same meta-analysis using the METAL software.

The primary gene-risk factor interaction analytic model included each SNP and risk factor (UV exposure or pigmentation traits) and an SNP × risk factor interaction term, as well as age, sex and the first five principal components as covariates:


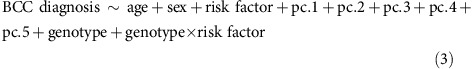


The logistic regression analyses within each platform were conducted with ProbABEL. Associations in each component GWAS set (Affymetrix, Illumina HumanHap series and Illumina Omni Express) were combined in an inverse-variance-weighted meta-analysis using the METAL software.

### Meta-analysis

For each SNP, meta-analysis was conducted to combine stage 1 and stage 2 results. Heterogeneity of per-SNP effect sizes in studies contributing to stage 1, stage 2 and the overall meta-analysis was assessed and fixed effects meta-analysis was conducted. The same method as in stage 1 was used to generate a set of index SNPs within each associated region. Top SNPs with *P*<5 × 10^−8^ were finally reported. All *R*^2^ and *D*' between individual SNPs were calculated based on the 1000 Genomes Pilot 1 data set, CEU Population (http://www.broadinstitute.org/mpg/snap/ldsearchpw.php)^62^.

### Proportion of familial relative risk

We have used the formula for calculating the proportion of FRR as outlined by the Cancer Oncological Gene-environment Study (http://www.nature.com/icogs/primer/common-variation-and-heritability-estimates-for-breast-ovarian-and-prostate-cancers/#70) as described previously^63^. The odds ratios derived from our meta-analysis of stages 1 and 2 are assumed to be relative risks. We estimated the proportion of the FRR explained by each SNP (FRR_snp_) as:


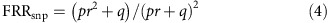


Here, the risk allele and alternative allele frequencies are *p* and *q*, respectively, and *r* is the odds ratio for the risk allele. Allele frequencies derived from the 1000 Genomes Project European population data. Assuming that the loci combine multiplicatively and are not in LD, the combined effect of all loci is given by:


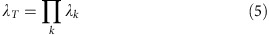


Here, the product is across all loci. The proportion of the familial relative risk attributable to the SNPs, on a log scale, is then given by:


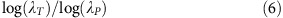


In this equation, *λ*_*P*_ is the familial relative risk observed in epidemiological studies. *λ*_*P*_ is 2.6-fold for BCC^60^,^64^.

### Regulatory function of novel variants

For each novel BCC susceptibility variant, we searched for evidence of regulatory function using recently updated HaploReg version 4 (http://www.broadinstitute.org/mammals/haploreg/haploreg.php)^65^,^66^. We queried each rsID and extracted data from ENCODE Project Consortium 2011–2012 on closest annotated gene, ChIP-Seq transcription factor binding, DNaseI hypersensitivity sites, and enhancer and promoter chromatin segmentation states^67^,^68^,^69^. We also extracted data from Roadmap Epigenomics Consortium 2015 on enhancer and promoter chromatin segmentation states, specifically using the following states: 15-state HMM, 25-state HMM, H3K4me1, H3K4me3, H3K27ac and H3K9ac^70^. We particularly focused on enhancer and promoter annotations that referenced normal human epidermal keratinocytes (NHEK) and primary foreskin keratinocytes. Finally, we used HaploReg v4 to extract eQTL data for each variant, as version 4 is updated with cis eQTL data from the GTEx pilot analysis and other studies^11^. We made special note of variants that were eQTLs in skin tissue.

### Gene expression analysis

Processed gene-expression data for BCC and normal skin (GSE53462 and GSE7553) were obtained from the GEO (http://www.ncbi.nlm.nih.gov/geo/)^71^,^72^,^73^. Fifteen BCC samples and five controls were included for GSE7553; for GSE53462, nine ‘classic-type' BCC samples were included, along with five controls. Each gene of interest was selected by its proximity to one of the 14 novel risk variants; however, if a variant was an eQTL in skin tissue for a more distant gene, then this gene was chosen instead. For each data set, Geo2R, which employs a linear-based model for microarray analysis, was utilized to compare gene expression between BCC and normal skin controls^74^. Significant results were defined as instances of differential gene expression (in BCC tissue relative to control) reaching *P*<0.05 in both data sets.

### Regional association and forest plots

Regional plots of –log10 (*P* values) were generated using Locus Zoom^75^. Where pairwise LD measures are given, using LD data from the March 2012 release of 1000 Genomes data. To preserve detail, results with *P*<10^−100^ are set to 10^−100^. In the plots, an ‘o' symbol indicates a genotyped SNP and a ‘+' indicates an imputed SNP. Colour indicates strength of LD with the index SNP. Forest plots were generated using the R forest plot package (https://cran.r-project.org/web/packages/forestplot/forestplot.pdf)^76^.

### Power calculations

Power was computed according to Freidlin *et al.*^77^. To account for misclassification, expected genotype frequencies in study cases were replaced with a mixture of genotype frequencies in true cases and in true controls. Power was plotted as a function of odds ratio for detecting a variant with minor allele frequency 0.1, based on the GWAS sample size and with hypothetical misclassification rates of 0, 10 and 20% (where the specified fraction of study cases are misclassified controls).

### Data availability

GWAS data from 23andMe and Nurses ‘Health/Health Professionals' Study have not been deposited in public repositories, as consent for this was not obtained in the study protocols. The pre-computed rankings and *P*-values for SNPs included in the stage 1 GWAS are available upon request by contacting D.A.H. at dhinds@23andMe.com. Pre-computed rankings and *P*-values for the top 10,000 SNPs included in the stage 2 GWAS are freely available by contacting www.channing.harvard.edu/nhs. Processed gene-expression data for BCC and normal skin are freely available from GEO (http://www.ncbi.nlm.nih.gov/geo/) using the accession numbers GSE53462 (ref. 72) and GSE7553 (ref. 73). Any additional data (beyond those included in the main text and Supplementary Information) that support the findings of this study are available from the corresponding author upon request.

## Additional information

**How to cite this article:** Chahal, H. S. *et al.* Genome-wide association study identifies 14 novel risk alleles associated with basal cell carcinoma. *Nat. Commun.* 7:12510 doi: 10.1038/ncomms12510 (2016).

## Supplementary Material

Supplementary Information Supplementary Figures 1-12 and Supplementary Tables 1-20

Peer review file

## Figures and Tables

**Table 1:** Interaction of 28 genome-wide significant BCC associations in stage 1 with melanoma.

SNP	Gene	*N*_effect_	*N*_SE_	*N*_*P*_	*Y*_effect_	*Y*_SE_	*Y*_*P*_	Int*P*
rs12203592	*IRF4*	0.397	0.017	5.4 × 10^−117^	0.454	0.060	4.5 × 10^−14^	0.040
rs1805007	*MC1R*	0.321	0.023	8.8 × 10^−41^	0.272	0.077	4.7 × 10^−4^	0.286
rs214785	*TGM3*	−0.197	0.017	1.7 × 10^−29^	−0.044	0.065	5.0 × 10^−1^	0.213
rs35407	*SLC45A2*	0.483	0.053	1.1 × 10^−22^	0.537	0.239	2.0 × 10^−2^	0.751
rs6059655	*RALY*	−0.219	0.024	5.7 × 10^−19^	−0.141	0.082	8.5 × 10^−2^	0.088
rs57142672	*RCC2*	0.128	0.014	1.2 × 10^−18^	0.214	0.052	4.4 × 10^−5^	0.612
rs73635312	*GATA3*	0.189	0.021	3.5 × 10^−20^	0.109	0.074	1.4 × 10^−1^	0.047
rs1126809	*TYR*	−0.126	0.015	2.3 × 10^−16^	−0.070	0.053	1.8 × 10^−1^	0.521
rs421284	*CLPTM1L*	0.138	0.014	1.1 × 10^−22^	0.046	0.050	3.5 × 10^−1^	0.783
rs2080303	*ALS2CR12/CASP8*	0.122	0.016	1.1 × 10^−14^	0.068	0.057	2.3 × 10^−1^	0.425
rs61824911	*RHOU*	0.139	0.016	9.3 × 10^−18^	−0.023	0.061	7.1 × 10^−1^	0.300
rs2116709	*FOXP1/EIF4E3*	−0.116	0.015	1.3 × 10^−14^	−0.061	0.054	2.6 × 10^−1^	0.136
rs12916300	*OCA2/HERC2*	0.142	0.019	9.7 × 10^−15^	−0.024	0.067	7.2 × 10^−1^	0.761
rs141115006	*RGS22*	−0.143	0.020	3.1 × 10^−13^	−0.077	0.071	2.8 × 10^−1^	0.819
rs10810657	*BNC2*	−0.108	0.014	7.2 × 10^−14^	−0.078	0.052	1.4 × 10^−1^	0.986
rs10093547	*ZFHX4*	0.219	0.031	5.1 × 10^−13^	0.147	0.106	1.6 × 10^−1^	0.755
rs191177147	*LPP*	0.114	0.016	6.3 × 10^−13^	0.009	0.057	8.8 × 10^−1^	0.517
rs9275642	*HLA-DQB1/DQA2*	−0.123	0.019	2.4 × 10^−11^	−0.080	0.065	2.2 × 10^−1^	0.346
rs7874604	*CDKN2B*	0.098	0.015	1.6 × 10^−10^	0.095	0.055	8.5 × 10^−2^	0.591
rs11170164	*KRT5*	0.163	0.025	1.2 × 10^−10^	0.134	0.094	1.6 × 10^−1^	0.303
rs73183643	*CUX1*	0.109	0.017	1.1 × 10^−10^	0.067	0.060	2.6 × 10^−1^	0.391
rs11993814	*ZBTB10*	−0.097	0.016	2.1 × 10^−9^	−0.146	0.058	1.2 × 10^−2^	0.413
rs2776353	*LINC00111*	−0.092	0.015	1.5 × 10^−9^	−0.063	0.055	2.5 × 10^−1^	0.623
rs1765871	*ATP11A*	−0.068	0.014	1.1 × 10^−6^	−0.145	0.052	4.8 × 10^−3^	0.981
rs10425559	*TICAM1/PLIN3*	0.086	0.014	2.7 × 10^−9^	0.004	0.052	9.3 × 10^−1^	0.458
rs1050529	*HLA-B*	−0.106	0.019	2.5 × 10^−8^	−0.096	0.069	1.7 × 10^−1^	0.486
rs7776701	*TNS3*	−0.081	0.014	6.9 × 10^−9^	0.041	0.050	4.1 × 10^−1^	0.711
rs78097823	*TTC28*	0.164	0.034	2.4 × 10^−6^	0.354	0.120	3.5 × 10^−3^	0.466

Results generated from logistic regression models fit separately in melanoma controls (N) and cases (Y). Includes *P*-value (Int *P*) from a likelihood ratio test for adding an interaction with melanoma status to the GWAS model. Of the 274,252 BCC controls, 3138 were melanoma cases and 268,282 were melanoma controls. Of the 12,945 BCC cases, 1,350 were melanoma cases and 11,465 were melanoma controls.

**Table 2:** Fourteen novel loci reaching genome-wide significance in two-stage GWAS of BCC.

SNP	Region	Gene	Maj/min	MAF (avg imp *r*^2^)	*P*	*OR*	*P*	*OR*	*P*	*OR*
rs2116709	3p13	*FOXP1*	A/T	0.40 (0.91)	7.9 × 10^−15^	0.89	6.1 × 10^−4^	0.91	2.3 × 10^−17^	0.90
rs10810657	9p22.2	*BNC2*	A/T	0.41 (0.98)	5.1 × 10^−14^	0.90	5.7 × 10^−5^	0.90	1.5 × 10^−17^	0.90
rs191177147	3q28	*LPP*	G/T	0.39 (0.80)	3.2 × 10^−12^	1.11	1.0 × 10^−3^	1.10	1.2 × 10^−14^	1.11
rs9275642	6p21.32	*HLA-DQA2*	C/T	0.21 (0.89)	1.2 × 10^−11^	0.89	2.7 × 10^−2^*	0.81	2.4 × 10^−12^	0.89
rs73183643	7q22.1	*CUX1*	G/A	0.24 (0.96)	8.5 × 10^−11^	0.90	2.3 × 10^−4^	0.89	1.5 × 10^−13^	0.90
rs11993814	8q21.13	*ZBTB10*	C/T	0.26 (1.0)	2.8 × 10^−10^	0.91	4.5 × 10^−2^	0.94	8.8 × 10^−11^	0.92
rs2776353	21q22.3	*LINC00111*	A/T	0.33 (0.96)	5.0 × 10^−10^	0.91	7.7 × 10^−4^	0.91	1.6 × 10^−12^	0.91
rs10425559	19p13.3	*PLIN3*	G/A	0.40 (0.97)	3.8 × 10^−9^	0.92	8.4 × 10^−1^	0.99	2.8 × 10^−8^	0.93
rs1050529	6p21.33	*HLA-B*	C/T	0.25 (0.71)	4.6 × 10^−9^	0.90	2.7 × 10^−1^†	0.89	2.6 × 10^−9^	0.90
rs7776701	7p12.3	*TNS3*	C/T	0.48 (0.98)	5.3 × 10^−9^	0.93	5.1 × 10^−1^	0.98	4.2 × 10^−8^	0.94
rs9267650	6p21.3	*NEU1*	A/T	0.05 (0.98)	2.4 × 10^−8^	1.18	2.0 × 10^−1^	1.09	1.1 × 10^−8^	1.17
rs7907606	10q24.3	*OBFC1*	T/G	0.17 (0.96)	7.4 × 10^−8^	1.10	2.4 × 10^−2^	1.08	4.7 × 10^−9^	1.10
rs4710154	6q27	*MIR3939*	A/T	0.32 (0.93)	8.1 × 10^−8^	1.08	4.3 × 10^−2^	1.06	1.1 × 10^−8^	1.08
rs2294214	6p22.3	*CASC15*	A/C	0.32 (0.95)	2.6 × 10^−5^	1.06	5.9 × 10^−5^	1.13	3.1 × 10^−8^	1.07

SNPs that met genome-wide significance (*P*<5 × 10^−8^, via logistic regression) in overall meta-analysis are listed; these SNPs have not been associated with BCC in previous GWAS reports. Additionally, we report genetic locus, nearest genes, major allele, minor allele, minor allele frequency (MAF) as calculated from stage 1 data, average imputation *r*^2^ (a measure of imputation quality) for stage 1, and odds ratio (OR) with *P-*value for each stage, calculated with respect to the minor allele. In stage 1, we analysed 12,945 BCC cases and 274,252 controls. Stage 2 included 4242 BCC cases and 12,802 controls from NHS. We then combined data from stage 1 and stage 2 (which resulted in 17,187 BCC cases and 287,054 controls) and performed fixed-effect meta-analysis. Statistics for effect heterogeneity (*P*_*het*_ and *I*^2^) are included in Supplementary Table 13. All subjects were from the US and of European ancestry.

^*^Genotyping results in stage 2.

^†^Genotyping results in stage 2. rs9266772 is used as proxy SNP for rs1050529 (*r*^2^=0.569, *D*′=0.771).

**Table 3:** Stage 2 subset analysis of novel BCC risk variant rs191177147 (3q28, *LPP*) in different hair colour subgroups.

Hair colour	*β*	s.e.	*P*	N	Int *P*-value
Whole set	0.097	0.03	1.0 × 10^−3^	17044	**0.0002**
Red and blonde	−0.008	0.062	9.0 × 10^−1^	2449	
Light brown	0.123	0.047	8.4 × 10^−3^	5793	
Dark brown and black	0.198	0.042	2.9 × 10^−6^	7611	

‘*β*' refers to effect size, ‘s.e.' to standard error, ‘*P*' to *P*-value (generated via logistic regression), ‘N' to sample size, and ‘Int' to interaction.

## References

[b1] Kauvar A. N. B. *et al.* Consensus for nonmelanoma skin cancer treatment: basal cell carcinoma, including a cost analysis of treatment methods. Dermatol. Surg. Off. Publ. Am. Soc. Dermatol. Surg. Al. 41, 550–571 (2015). 10.1097/DSS.0000000000000296 25868035

[b2] Stacey S. N. *et al.* Common variants on 1p36 and 1q42 are associated with cutaneous basal cell carcinoma but not with melanoma or pigmentation traits. Nat. Genet. 40, 1313–1318 (2008). 18849993 10.1038/ng.234

[b3] Rafnar T. *et al.* Sequence variants at the TERT-CLPTM1L locus associate with many cancer types. Nat. Genet. 41, 221–227 (2009). 19151717 10.1038/ng.296 PMC4525478

[b4] Nan H. *et al.* Genome-wide association study identifies novel alleles associated with risk of cutaneous basal cell carcinoma and squamous cell carcinoma. Hum. Mol. Genet. 20, 3718–3724 (2011). 21700618 10.1093/hmg/ddr287 PMC3159556

[b5] Stacey S. N. *et al.* A germline variant in the TP53 polyadenylation signal confers cancer susceptibility. Nat. Genet. 43, 1098–1103 (2011). 21946351 10.1038/ng.926 PMC3263694

[b6] Stacey S. N. *et al.* Germline sequence variants in TGM3 and RGS22 confer risk of basal cell carcinoma. Hum. Mol. Genet. 23, 3045–3053 (2014). 24403052 10.1093/hmg/ddt671 PMC4014188

[b7] Stacey S. N. *et al.* New basal cell carcinoma susceptibility loci. Nat. Commun. 6, 6825 (2015). 25855136 10.1038/ncomms7825 PMC4403348

[b8] Mangino M. *et al.* Genome-wide meta-analysis points to CTC1 and ZNF676 as genes regulating telomere homeostasis in humans. Hum. Mol. Genet. 21, 5385–5394 (2012). 23001564 10.1093/hmg/dds382 PMC3510758

[b9] Chen L.-Y., Redon S. & Lingner J. The human CST complex is a terminator of telomerase activity. Nature 488, 540–544 (2012). 22763445 10.1038/nature11269

[b10] Levy D. *et al.* Genome-wide association identifies OBFC1 as a locus involved in human leukocyte telomere biology. Proc. Natl Acad. Sci. USA 107, 9293–9298 (2010). 20421499 10.1073/pnas.0911494107 PMC2889047

[b11] GTEx Consortium. Human genomics. The Genotype-Tissue Expression (GTEx) pilot analysis: multitissue gene regulation in humans. Science 348, 648–660 (2015). 25954001 10.1126/science.1262110 PMC4547484

[b12] Anic G. M. *et al.* Telomere length and risk of melanoma, squamous cell carcinoma, and basal cell carcinoma. Cancer Epidemiol. 37, 434–439 (2013). 23523330 10.1016/j.canep.2013.02.010 PMC3679277

[b13] Remmers E. F. *et al.* Genome-wide association study identifies variants in the MHC class I, IL10, and IL23R-IL12RB2 regions associated with Behçet's disease. Nat. Genet. 42, 698–702 (2010). 20622878 10.1038/ng.625 PMC2923807

[b14] Zhu K.-J. *et al.* Psoriasis regression analysis of MHC loci identifies shared genetic variants with vitiligo. PloS One 6, e23089 (2011). 22125590 10.1371/journal.pone.0023089 PMC3220662

[b15] Mendez R. *et al.* HLA and melanoma: multiple alterations in HLA class I and II expression in human melanoma cell lines from ESTDAB cell bank. Cancer Immunol. Immunother. CII 58, 1507–1515 (2009). 19340423 10.1007/s00262-009-0701-z PMC11030131

[b16] Méndez R. *et al.* Characterization of HLA class I altered phenotypes in a panel of human melanoma cell lines. Cancer Immunol. Immunother. CII 57, 719–729 (2008). 17934731 10.1007/s00262-007-0411-3 PMC11030649

[b17] Coenen M. J. H. *et al.* Common and different genetic background for rheumatoid arthritis and coeliac disease. Hum. Mol. Genet. 18, 4195–4203 (2009). 19648290 10.1093/hmg/ddp365

[b18] Hinks A. *et al.* Investigation of type 1 diabetes and coeliac disease susceptibility loci for association with juvenile idiopathic arthritis. Ann. Rheum. Dis. 69, 2169–2172 (2010). 20647273 10.1136/ard.2009.126938 PMC3002762

[b19] Jin Y. *et al.* Variant of TYR and autoimmunity susceptibility loci in generalized vitiligo. N. Engl. J. Med. 362, 1686–1697 (2010). 20410501 10.1056/NEJMoa0908547 PMC2891985

[b20] Hinds D. A. *et al.* A genome-wide association meta-analysis of self-reported allergy identifies shared and allergy-specific susceptibility loci. Nat. Genet. 45, 907–911 (2013). 23817569 10.1038/ng.2686 PMC3753407

[b21] Grunewald T. G., Pasedag S. M. & Butt E. Cell adhesion and transcriptional activity—defining the role of the novel protooncogene LPP. Transl. Oncol. 2, 107–116 (2009). 19701494 10.1593/tlo.09112 PMC2730141

[b22] Weber A. *et al.* Proapoptotic signalling through Toll-like receptor-3 involves TRIF-dependent activation of caspase-8 and is under the control of inhibitor of apoptosis proteins in melanoma cells. Cell Death Differ. 17, 942–951 (2010). 20019748 10.1038/cdd.2009.190

[b23] Burguete A. S., Sivars U. & Pfeffer S. Purification and analysis of TIP47 function in Rab9-dependent mannose 6-phosphate receptor trafficking. Methods Enzymol. 403, 357–366 (2005). 16473602 10.1016/S0076-6879(05)03031-4

[b24] Than G. N. *et al.* Overexpression of placental tissue protein 17b/TIP47 in cervical dysplasias and cervical carcinoma. Anticancer Res. 21, 639–642 (2001). 11299819

[b25] Abdulkhalek S. *et al.* Neu1 sialidase and matrix metalloproteinase-9 cross-talk is essential for Toll-like receptor activation and cellular signaling. J. Biol. Chem. 286, 36532–36549 (2011). 21873432 10.1074/jbc.M111.237578 PMC3196117

[b26] Antonicelli F., Bellon G., Lorimier S. & Hornebeck W. Role of the elastin receptor complex (S-Gal/Cath-A/Neu-1) in skin repair and regeneration. Wound Repair Regen. Off. Publ. Wound Heal. Soc. Eur. Tissue Repair Soc. 17, 631–638 (2009). 10.1111/j.1524-475X.2009.00525.x 19769716

[b27] Kwak J. E., Son M.-Y., Son Y. S., Son M. J. & Cho Y. S. Biochemical and molecular characterization of novel mutations in GLB1 and NEU1 in patient cells with lysosomal storage disorders. Biochem. Biophys. Res. Commun. 457, 554–560 (2015). 25600812 10.1016/j.bbrc.2015.01.023

[b28] Ren L.-R. *et al.* Effects of sialidase NEU1 siRNA on proliferation, apoptosis, and invasion in human ovarian cancer. Mol. Cell. Biochem. 411, 213–219 (2016). 26463994 10.1007/s11010-015-2583-z

[b29] Seldin M. F. *et al.* Genome-wide association study of late-onset myasthenia gravis: confirmation of TNFRSF11A, and identification of ZBTB10 and three distinct HLA associations. Mol. Med. Camb. Mass. 21, 769–781 (2015). 10.2119/molmed.2015.00232 PMC4749491 26562150

[b30] Tone M., Powell M. J., Tone Y., Thompson S. A. & Waldmann H. IL-10 gene expression is controlled by the transcription factors Sp1 and Sp3. J. Immunol. Baltim. Md 1950 165, 286–291 (2000). 10.4049/jimmunol.165.1.286 10861063

[b31] Tillotson L. G. RIN ZF, a novel zinc finger gene, encodes proteins that bind to the CACC element of the gastrin promoter. J. Biol. Chem. 274, 8123–8128 (1999). 10075714 10.1074/jbc.274.12.8123

[b32] Lai Y. *et al.* The microRNA-27a: ZBTB10-specificity protein pathway is involved in follicle stimulating hormone-induced VEGF, Cox2 and survivin expression in ovarian epithelial cancer cells. Int. J. Oncol. 42, 776–784 (2013). 23254909 10.3892/ijo.2012.1743

[b33] Takayama K.-I. *et al.* Integrative analysis of FOXP1 function reveals a tumor-suppressive effect in prostate cancer. Mol. Endocrinol. Baltim. Md 28, 2012–2024 (2014). 10.1210/me.2014-1171 PMC5414778 25329375

[b34] Levine D. M. *et al.* A genome-wide association study identifies new susceptibility loci for esophageal adenocarcinoma and Barrett's esophagus. Nat. Genet. 45, 1487–1493 (2013). 24121790 10.1038/ng.2796 PMC3840115

[b35] Koon H. B., Ippolito G. C., Banham A. H. & Tucker P. W. FOXP1: a potential therapeutic target in cancer. Expert Opin. Ther. Targets 11, 955–965 (2007). 17614763 10.1517/14728222.11.7.955 PMC4282158

[b36] Ripka S. *et al.* Glutamate receptor GRIA3—target of CUX1 and mediator of tumor progression in pancreatic cancer. Neoplasia N. Y. N 12, 659–667 (2010). 10.1593/neo.10486 PMC2915410 20689760

[b37] Fan X. *et al.* The transcription factor CUTL1 is associated with proliferation and prognosis in malignant melanoma. Melanoma Res. 24, 198–206 (2014). 24686420 10.1097/CMR.0000000000000064

[b38] Al Olama A. A. *et al.* A meta-analysis of 87,040 individuals identifies 23 new susceptibility loci for prostate cancer. Nat. Genet. 46, 1103–1109 (2014). 25217961 10.1038/ng.3094 PMC4383163

[b39] Qian X. *et al.* The Tensin-3 protein, including its SH2 domain, is phosphorylated by Src and contributes to tumorigenesis and metastasis. Cancer Cell 16, 246–258 (2009). 19732724 10.1016/j.ccr.2009.07.031 PMC3293497

[b40] Chen X. *et al.* RNASET2 tag SNP but not CCR6 polymorphisms is associated with autoimmune thyroid diseases in the Chinese Han population. BMC Med. Genet. 16, 11 (2015). 25928629 10.1186/s12881-015-0150-9 PMC4422281

[b41] Lualdi M. *et al.* Pleiotropic modes of action in tumor cells of RNASET2, an evolutionary highly conserved extracellular RNase. Oncotarget 6, 7851–7865 (2015). 25797262 10.18632/oncotarget.3490 PMC4480721

[b42] Monti L. *et al.* RNASET2 as a tumor antagonizing gene in a melanoma cancer model. Oncol. Res. 17, 69–74 (2008). 18543608 10.3727/096504008784523658

[b43] Wang Q. *et al.* Stress-induced RNASET2 overexpression mediates melanocyte apoptosis via the TRAF2 pathway in vitro. Cell Death Dis. 5, e1022 (2014). 24457966 10.1038/cddis.2013.539 PMC4040706

[b44] Sand M. *et al.* Long-noncoding RNAs in basal cell carcinoma. Tumour Biol. J. Int. Soc. Oncodevelopmental Biol. Med. 37, 1–14 (2016). 10.1007/s13277-016-4927-z 26861560

[b45] Lessard L. *et al.* The CASC15 long intergenic noncoding RNA locus is involved in melanoma progression and phenotype switching. J. Invest. Dermatol. 135, 2464–2474 (2015). 26016895 10.1038/jid.2015.200 PMC4567947

[b46] Visser M., Palstra R.-J. & Kayser M. Human skin color is influenced by an intergenic DNA polymorphism regulating transcription of the nearby BNC2 pigmentation gene. Hum. Mol. Genet. 23, 5750–5762 (2014). 24916375 10.1093/hmg/ddu289

[b47] Jacobs L. C. *et al.* A genome-wide association study identifies the skin color genes IRF4, MC1R, ASIP, and BNC2 influencing facial pigmented spots. J. Invest. Dermatol. 135, 1735–1742 (2015). 25705849 10.1038/jid.2015.62

[b48] Eriksson N. *et al.* Web-based, participant-driven studies yield novel genetic associations for common traits. PLoS Genet. 6, e1000993 (2010). 20585627 10.1371/journal.pgen.1000993 PMC2891811

[b49] Durand E. Y., Do C. B., Mountain J. L. & Macpherson J. M. Ancestry Composition: A Novel, Efficient Pipeline for Ancestry Deconvolution (2014).

[b50] Henn B. M. *et al.* Cryptic distant relatives are common in both isolated and cosmopolitan genetic samples. PloS One 7, e34267 (2012). 22509285 10.1371/journal.pone.0034267 PMC3317976

[b51] 1000 Genomes Project Consortium. *et al.* A map of human genome variation from population-scale sequencing. Nature 467, 1061–1073 (2010). 20981092 10.1038/nature09534 PMC3042601

[b52] Browning S. R. & Browning B. L. Rapid and accurate haplotype phasing and missing-data inference for whole-genome association studies by use of localized haplotype clustering. Am. J. Hum. Genet. 81, 1084–1097 (2007). 17924348 10.1086/521987 PMC2265661

[b53] Fuchsberger C., Abecasis G. R. & Hinds D. A. minimac2: faster genotype imputation. Bioinforma. Oxf. Engl. 31, 782–784 (2015). 10.1093/bioinformatics/btu704 PMC4341061 25338720

[b54] Jorgenson E. *et al.* A genome-wide association study identifies four novel susceptibility loci underlying inguinal hernia. Nat. Commun. 6, 10130 (2015). 26686553 10.1038/ncomms10130 PMC4703831

[b55] Hu Y. *et al.* GWAS of 89,283 individuals identifies genetic variants associated with self-reporting of being a morning person. Nat. Commun. 7, 10448 (2016). 26835600 10.1038/ncomms10448 PMC4740817

[b56] Yu K. *et al.* Population substructure and control selection in genome-wide association studies. PLoS One 3, e2551 (2008). 18596976 10.1371/journal.pone.0002551 PMC2432498

[b57] Colditz G. A. *et al.* Validation of questionnaire information on risk factors and disease outcomes in a prospective cohort study of women. Am. J. Epidemiol. 123, 894–900 (1986). 3962971 10.1093/oxfordjournals.aje.a114319

[b58] Hunter D. J. *et al.* Risk factors for basal cell carcinoma in a prospective cohort of women. Ann. Epidemiol. 1, 13–23 (1990). 1669486 10.1016/1047-2797(90)90015-k

[b59] van Dam R. M. *et al.* Risk factors for basal cell carcinoma of the skin in men: results from the health professionals follow-up study. Am. J. Epidemiol. 150, 459–468 (1999). 10472945 10.1093/oxfordjournals.aje.a010034

[b60] Han J., Colditz G. A. & Hunter D. J. Risk factors for skin cancers: a nested case-control study within the Nurses' Health Study. Int. J. Epidemiol. 35, 1514–1521 (2006). 16943234 10.1093/ije/dyl197

[b61] Price A. L. *et al.* Principal components analysis corrects for stratification in genome-wide association studies. Nat. Genet. 38, 904–909 (2006). 16862161 10.1038/ng1847

[b62] Johnson A. D. *et al.* SNAP: a web-based tool for identification and annotation of proxy SNPs using HapMap. Bioinforma. Oxf. Engl. 24, 2938–2939 (2008). 10.1093/bioinformatics/btn564 PMC2720775 18974171

[b63] Law M. H. *et al.* Genome-wide meta-analysis identifies five new susceptibility loci for cutaneous malignant melanoma. Nat. Genet. 47, 987–995 (2015). 26237428 10.1038/ng.3373 PMC4557485

[b64] Berlin N. L. *et al.* Family history of skin cancer is associated with early-onset basal cell carcinoma independent of MC1R genotype. Cancer Epidemiol. 39, 1078–1083 (2015). 26381319 10.1016/j.canep.2015.09.005 PMC4679454

[b65] Wang J. *et al.* Sequence features and chromatin structure around the genomic regions bound by 119 human transcription factors. Genome Res. 22, 1798–1812 (2012). 22955990 10.1101/gr.139105.112 PMC3431495

[b66] Ward L. D. & Kellis M. HaploReg: a resource for exploring chromatin states, conservation, and regulatory motif alterations within sets of genetically linked variants. Nucleic Acids Res. 40, D930–D934 (2012). 22064851 10.1093/nar/gkr917 PMC3245002

[b67] Ernst J. *et al.* Mapping and analysis of chromatin state dynamics in nine human cell types. Nature 473, 43–49 (2011). 21441907 10.1038/nature09906 PMC3088773

[b68] ENCODE Project Consortium. A user's guide to the encyclopedia of DNA elements (ENCODE). PLoS Biol. 9, e1001046 (2011). 21526222 10.1371/journal.pbio.1001046 PMC3079585

[b69] ENCODE Project Consortium. An integrated encyclopedia of DNA elements in the human genome. Nature 489, 57–74 (2012). 22955616 10.1038/nature11247 PMC3439153

[b70] Chadwick L. H. The NIH Roadmap Epigenomics Program data resource. Epigenomics 4, 317–324 (2012). 22690667 10.2217/epi.12.18 PMC3381455

[b71] Edgar R., Domrachev M. & Lash A. E. Gene Expression Omnibus: NCBI gene expression and hybridization array data repository. Nucleic Acids Res. 30, 207–210 (2002). 11752295 10.1093/nar/30.1.207 PMC99122

[b72] Jee B. A. *et al.* Molecular classification of basal cell carcinoma of skin by gene expression profiling. Mol. Carcinog. 54, 1605–1612 (2015). 25328065 10.1002/mc.22233

[b73] Riker A. I. *et al.* The gene expression profiles of primary and metastatic melanoma yields a transition point of tumor progression and metastasis. BMC Med. Genomics 1, 13 (2008). 18442402 10.1186/1755-8794-1-13 PMC2408576

[b74] Davis S. & Meltzer P. S. GEOquery: a bridge between the Gene Expression Omnibus (GEO) and BioConductor. Bioinforma. Oxf. Engl. 23, 1846–1847 (2007). 10.1093/bioinformatics/btm254 17496320

[b75] Pruim R. J. *et al.* LocusZoom: regional visualization of genome-wide association scan results. Bioinforma. Oxf. Engl. 26, 2336–2337 (2010). 10.1093/bioinformatics/btq419 PMC2935401 20634204

[b76] Gordon M. Package ‘forestplot'. Advanced Forest Plot Using ‘grid' Graphics (2016).

[b77] Freidlin B., Zheng G., Li Z. & Gastwirth J. L. Trend tests for case-control studies of genetic markers: power, sample size and robustness. Hum. Hered. 53, 146–152 (2002). 12145550 10.1159/000064976

