# Supplementary material for: Genome-wide association study identifies 14 novel risk alleles associated with basal cell carcinoma
Source: Nat Commun. 2016 Aug 19;7:12510. doi: 10.1038/ncomms12510 (PMC4992160; doi:10.1038/ncomms12510)
Supplement: Supplementary Information — Supplementary Figures 1-12 and Supplementary Tables 1-20 [file ncomms12510-s1.pdf]

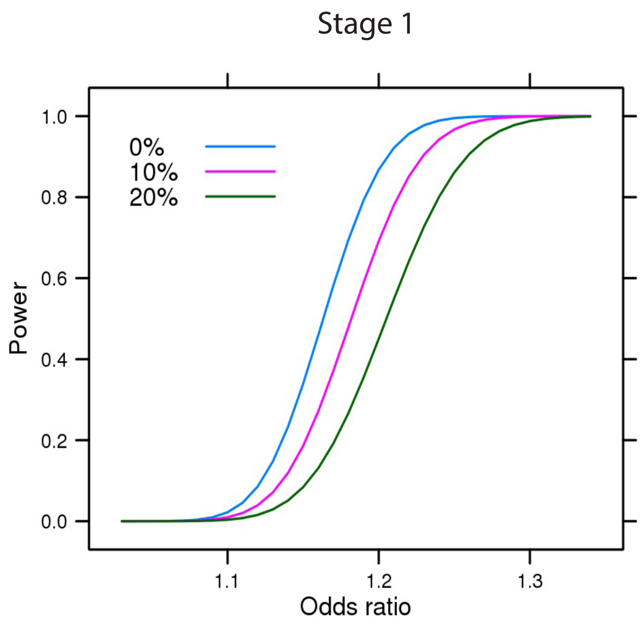

Power	Percent misclassified cases *0%*	*10%*	*20%*
0.2	1.14	1.15	1.17
0.5	1.16	1.18	1.21
0.8	1.19	1.21	1.24

Supplementary Figure 1. Power to detect a variant with misclassifications rates between 0 and 20%. The top graph depicts power as a function of odds ratio for detecting a variant with minor allele frequency 0.1, with misclassification rates of 0%, 10%, and 20% (where the specified fraction of study cases are misclassified controls). The table (bottom) shows odds ratios required to achieve the specified power for a variant with minor allele frequency 0.1, based on the GWAS sample size, for misclassification rates of 0%, 10%, and 20%. To account for misclassification, expected genotype frequencies in study cases were replaced with a mixture of genotype frequencies in true cases and in true controls.

Stage 1, genotyped SNPs only

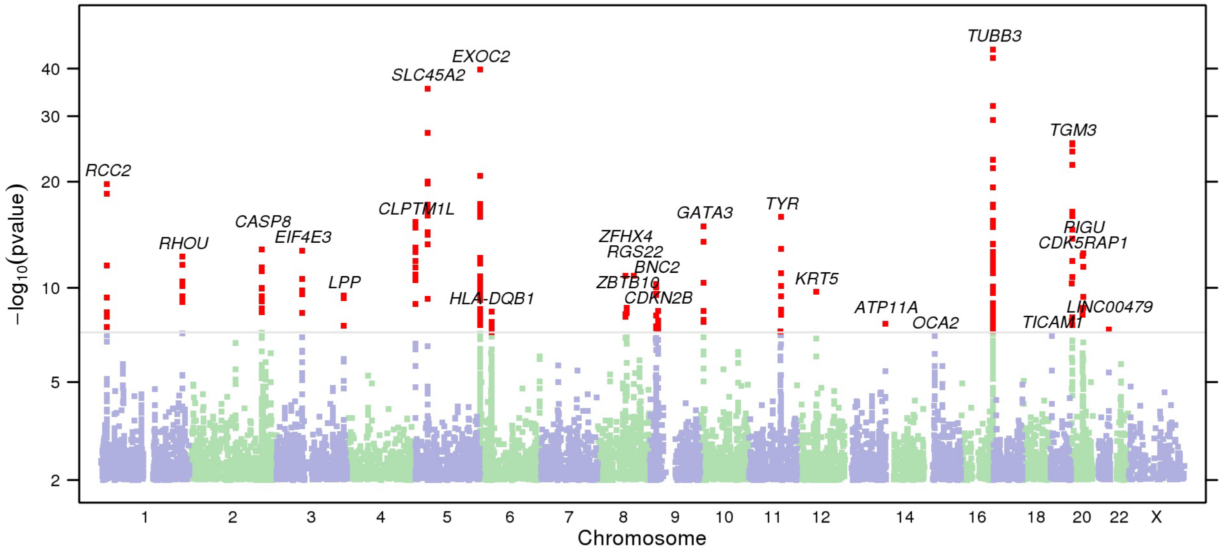

Supplementary Figure 2. Manhattan plot of stage 1 showing associations between genotyped SNPs and BCC. Total stage 1 GWAS analysis included 12,945 cases and 274,252 controls. Loci with smallest $P<10^{-6}$ (generated via logistic regression) are labeled with the name of the nearest gene; for clarity, only the 25 most significant loci are labeled. Positions with $P<5\times10^{-8}$ (genome-wide significance) are shown in red.

Stage 1, genotyped and imputed SNPs

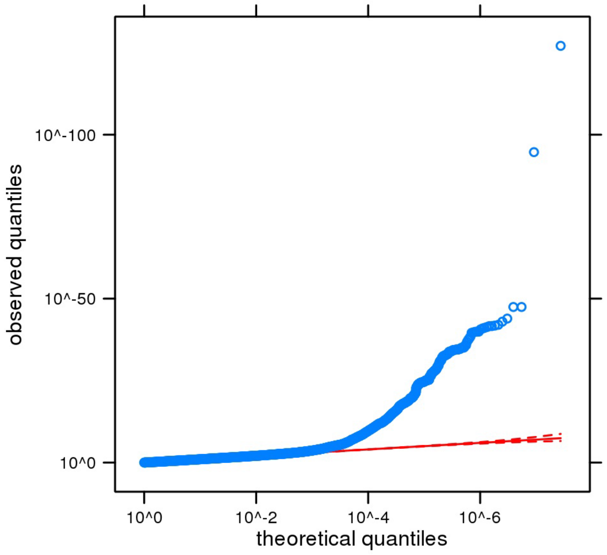

Supplementary Figure 3. QQ plot of observed versus expected quantiles for the BCC GWAS stage 1 *P* values (generated via logistic regression), plotted on a log scale. The null hypothesis states that the expected distribution of *P* values is uniform. Here, the observed *P* values follow the null distribution for large *P* values (*P*>0.01) but then diverge for small *P* values. The solid red line has a slope 1 and the dashed red lines represent a 95% confidence interval, assuming the test results are independent. The test statistics in the plot have been adjusted for genomic control.

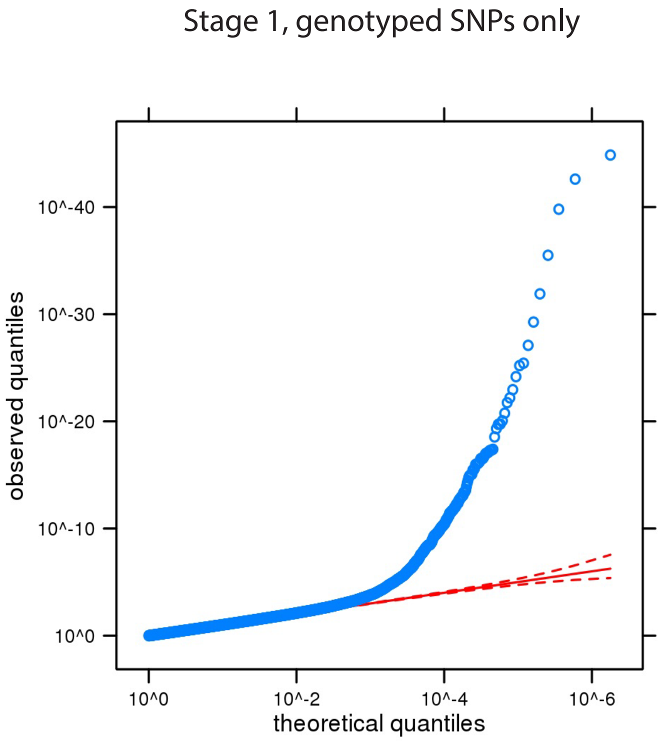

Supplementary Figure 4. QQ plot of observed versus expected quantiles for stage 1 *P* values, plotted on a log scale, when only genotyped SNPs are included. The dashed line has a slope 1. (Genomic inflation factor, λ= 1.091).

Stage 1, excluding 28 genome-wide significant regions

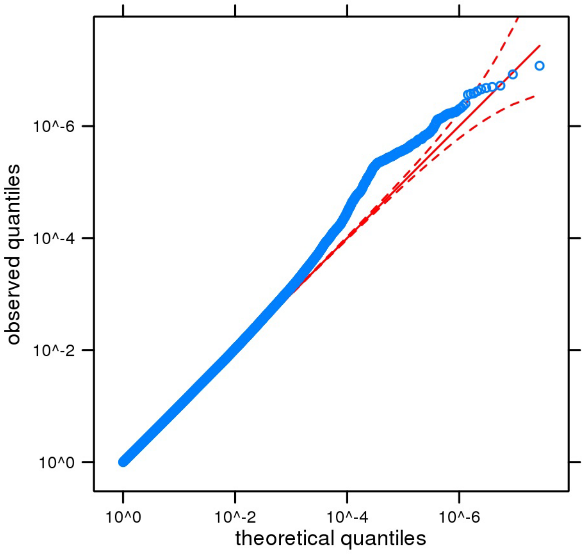

Supplementary Figure 5. QQ plot of observed versus expected quantiles for stage 1 *P* values, plotted on a log scale, excluding 28 genome-wide significant loci from stage 1 and their surrounding 500kb interval. The dashed line has a slope 1.

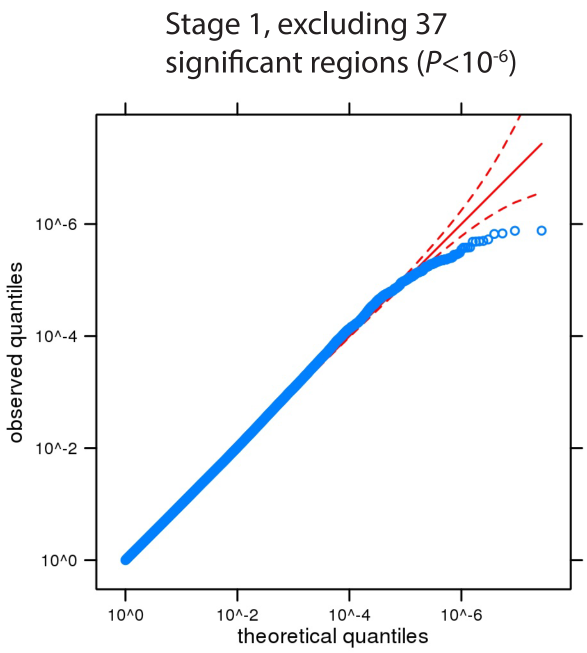

Supplementary Figure 6. QQ plot of observed versus expected quantiles for stage 1 *P* values, plotted on a log scale, excluding 37 significant loci ($P<10^{-6}$) from stage 1 and their surrounding 500kb interval. The dashed line has a slope 1.

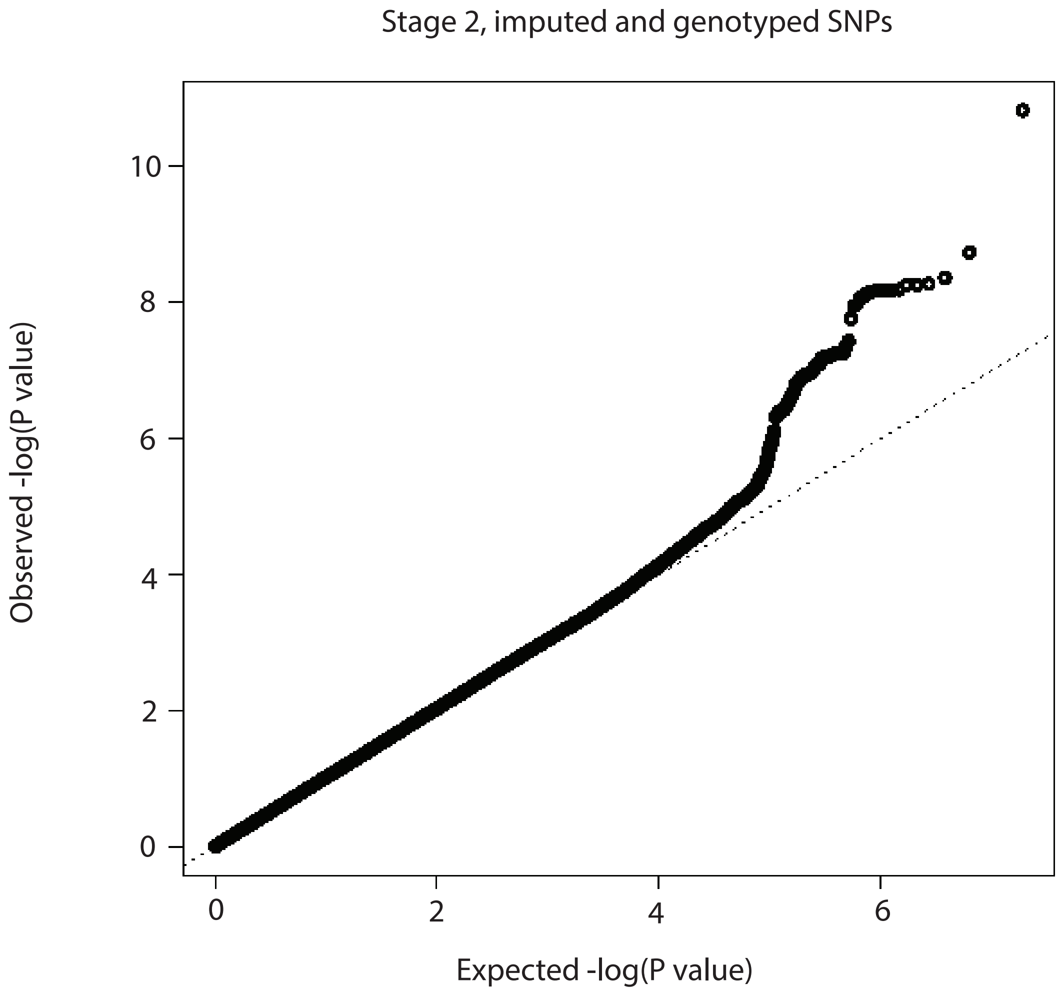

Supplementary Figure 7. QQ plot of observed versus expected quantiles for stage 2 *P* values, plotted on a log scale. The dashed line has a slope 1. (Genomic inflation factor, λ=1.023).

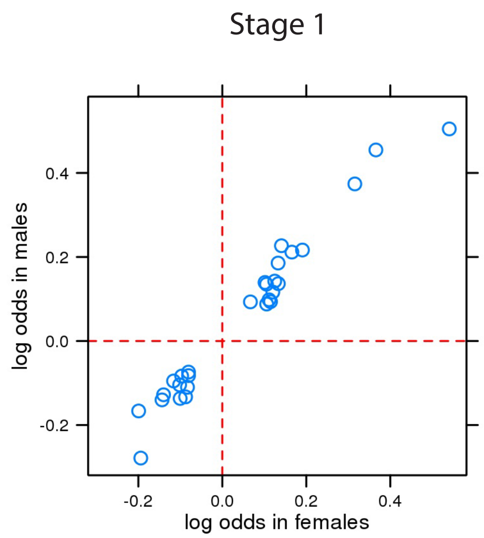

Supplementary Figure 8. Effect sizes estimated in men versus women, for the 28 genome-wide significant loci from stage 1.

Stage 1

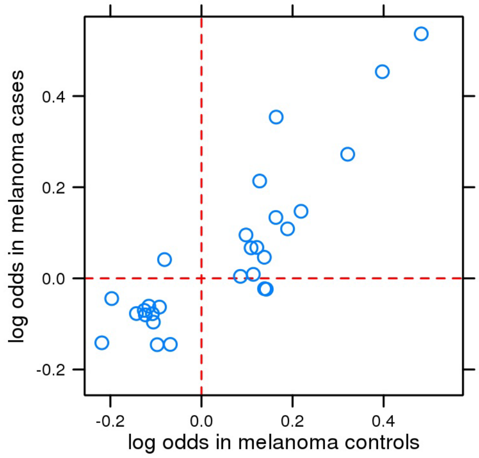

Supplementary Figure 9. Effect sizes estimated in individuals with melanoma versus individuals without melanoma, for the 28 genome-wide significant loci from stage 1.

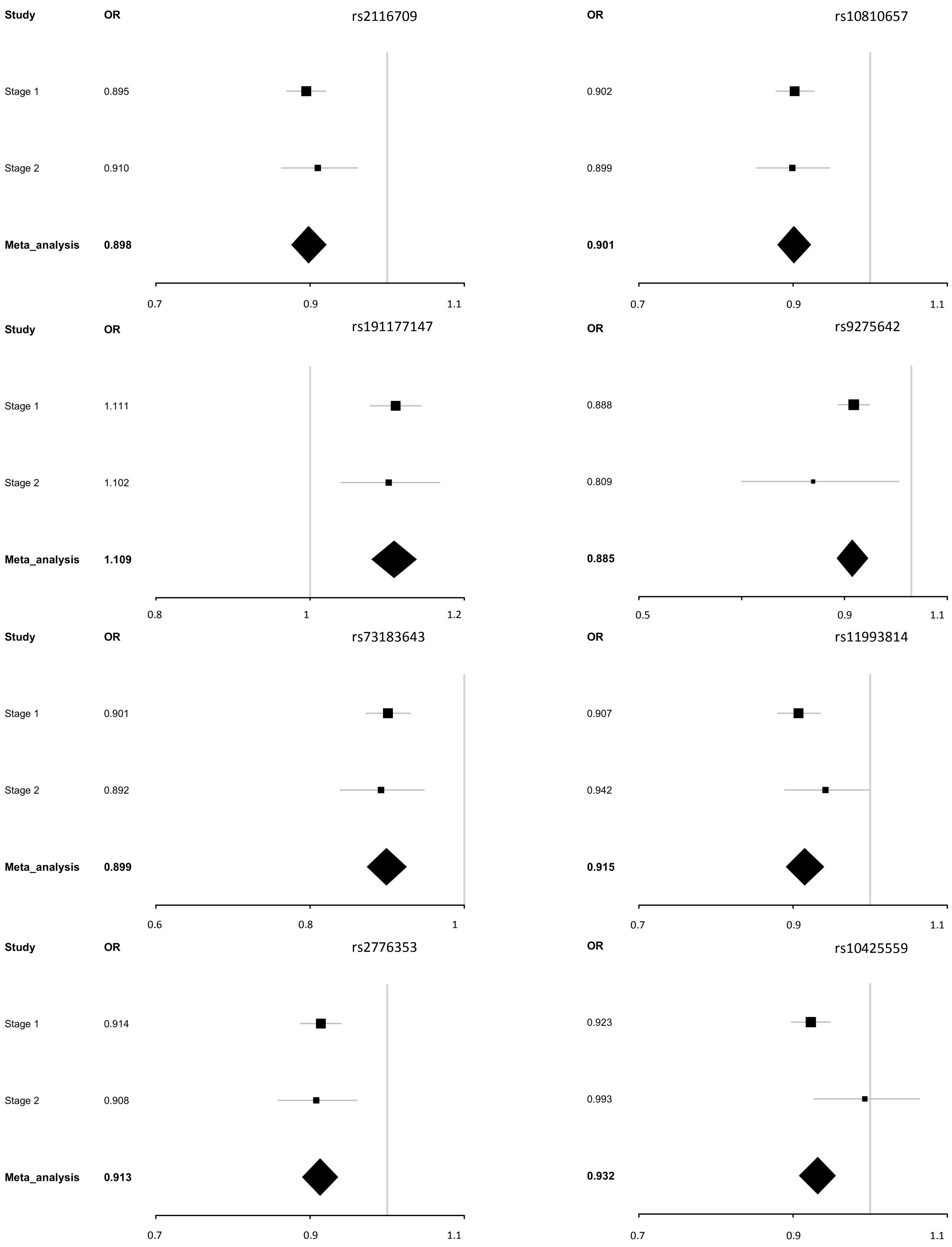

Supplementary Figure 10

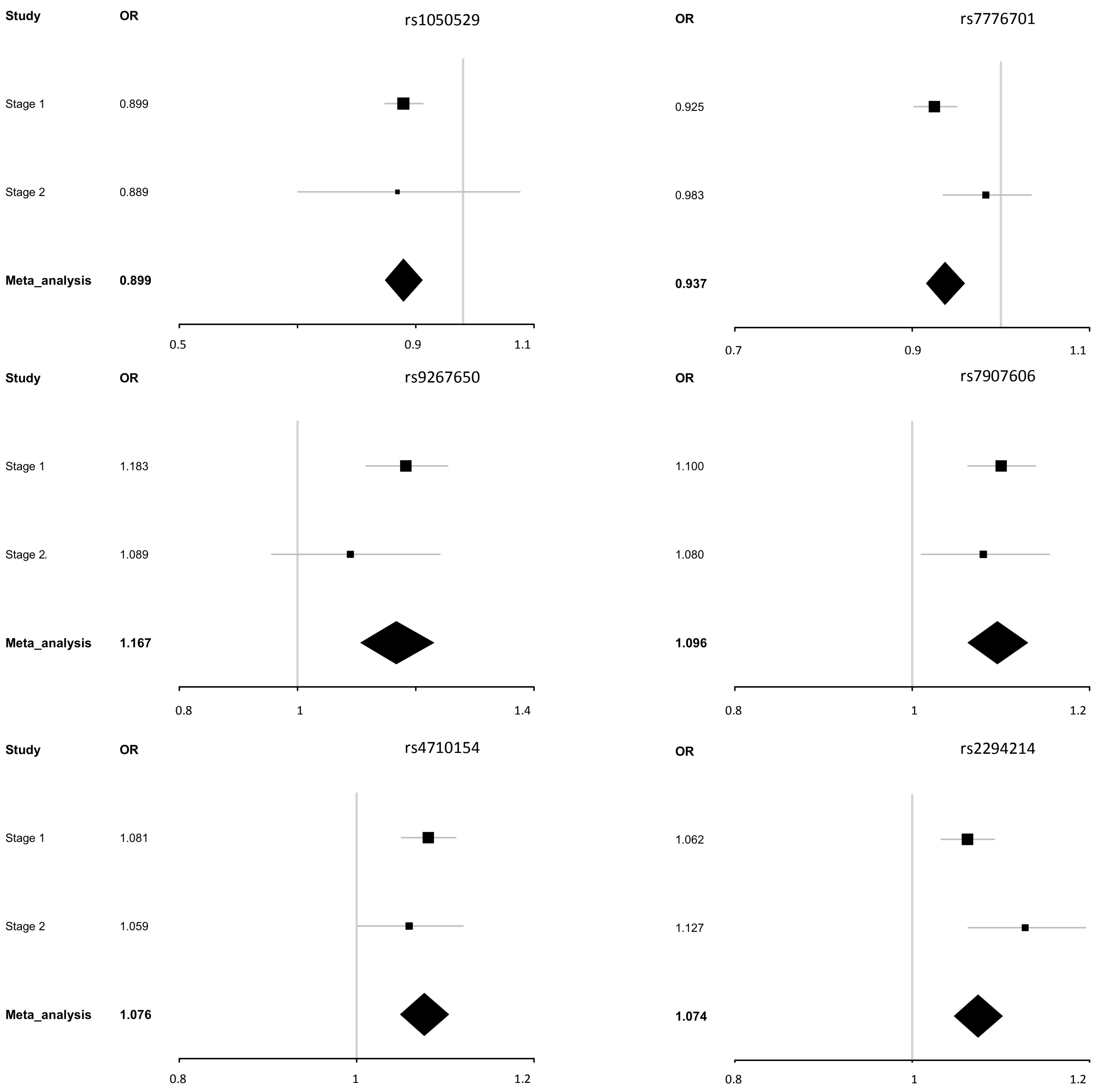

Supplementary Figure 10. Forest plots for each of the 14 novel SNPs reaching genome-wide significance for association with BCC (via logistic regression). For all plots, x-axis displays odds ratio (OR) values and gray vertical lines represent an odds ratio (OR) of 1. Each black square indicates OR from the corresponding stage and horizontal gray lines represent 95% CI. The center of each diamond represents the OR for the overall meta-analysis, and the width of each diamond represents the 95% CI.

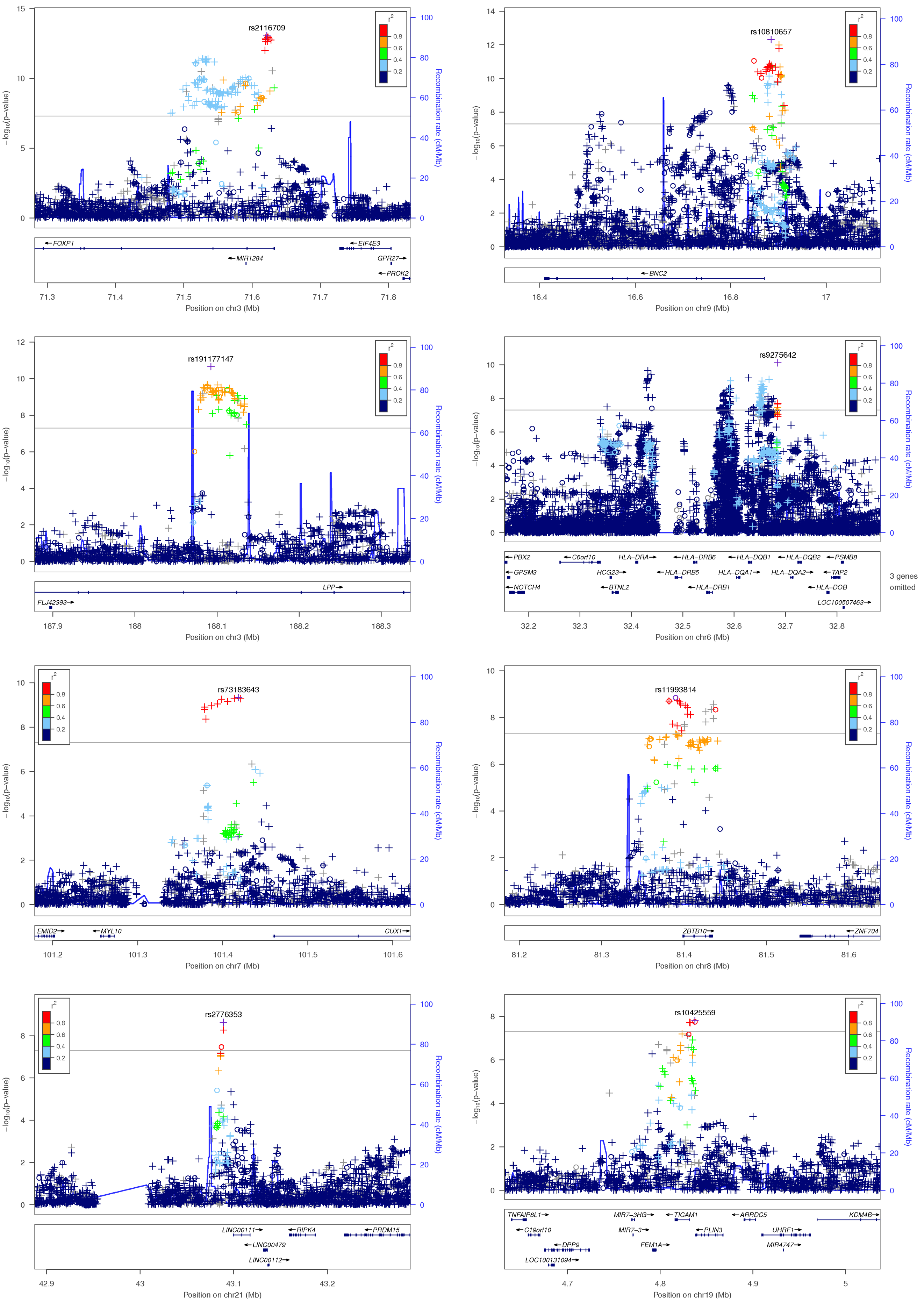
rs2116709
rs10810657
rs191177147
rs9275642
rs73183643
rs11993814
rs2776353
rs10425559
r2
0.8
0.6
0.4
0.2
−log10(p−value)
Recombination rate (cM/Mb)
FOXP1
MIR1284
EIF4E3
GPR27
PROK2
Position on chr3 (Mb)
BNC2
Position on chr9 (Mb)
LPP
FLJ42393
Position on chr3 (Mb)
PBX2
GPSM3
NOTCH4
C6orf10
HCG23
BTNL2
HLA−DRA
HLA−DRB6
HLA−DRB5
HLA−DRB1
HLA−DQB1
HLA−DQA1
HLA−DQB2
HLA−DQA2
HLA−DOB
PSMB8
TAP2
LOC100507463
3 genes omitted
Position on chr6 (Mb)
EMID2
MYL10
CUX1
Position on chr7 (Mb)
ZBTB10
ZNF704
Position on chr8 (Mb)
LINC00111
LINC00479
LINC00112
RIPK4
PRDM15
Position on chr21 (Mb)
TNFAIP8L1
C19orf10
DPP9
LOC100131094
MIR7−3HG
MIR7−3
FEM1A
TICAM1
PLIN3
ARRDC5
UHRF1
MIR4747
KDM4B
Position on chr19 (Mb)

Supplementary Figure 11. Regional association plots for the first 8 of 14 novel, genome-wide significant BCC susceptibility loci are shown. Left to right, beginning from top left: 3p13 (rs2116709, *FOXP1*), 9p22.2 (rs10810657, near *BNC2*), 3q28 (rs191177147, *LPP*), 6p21.32 (rs9275642, *HLA-DQA2*), 7q22.1 (rs73183643, *CUX1*), 8q21.13 (rs11993814, *ZBTB10*), 21q22.3 (rs2776353, *LINC00111*), 19p13.3.2 (rs10425559, *PLIN3*). Each plot displays $-\log_{10}(P \text{ value})$ versus genomic position based on stage 1 logistic regression association testing. The color scale indicates strength of linkage disequilibrium (r^2) for nearby SNPs, with respect to the index SNP. To preserve detail, results with $P<10^{-100}$ are set to 10^{-100}. The "o" and "+" symbols represent genotyped and imputed SNPs, respectively. Recombination rates, in cM/Mb, are also plotted (navy blue lines). These plots were generated via LocusZoom, using LD data from the March 2012 release of 1000 Genomes data.

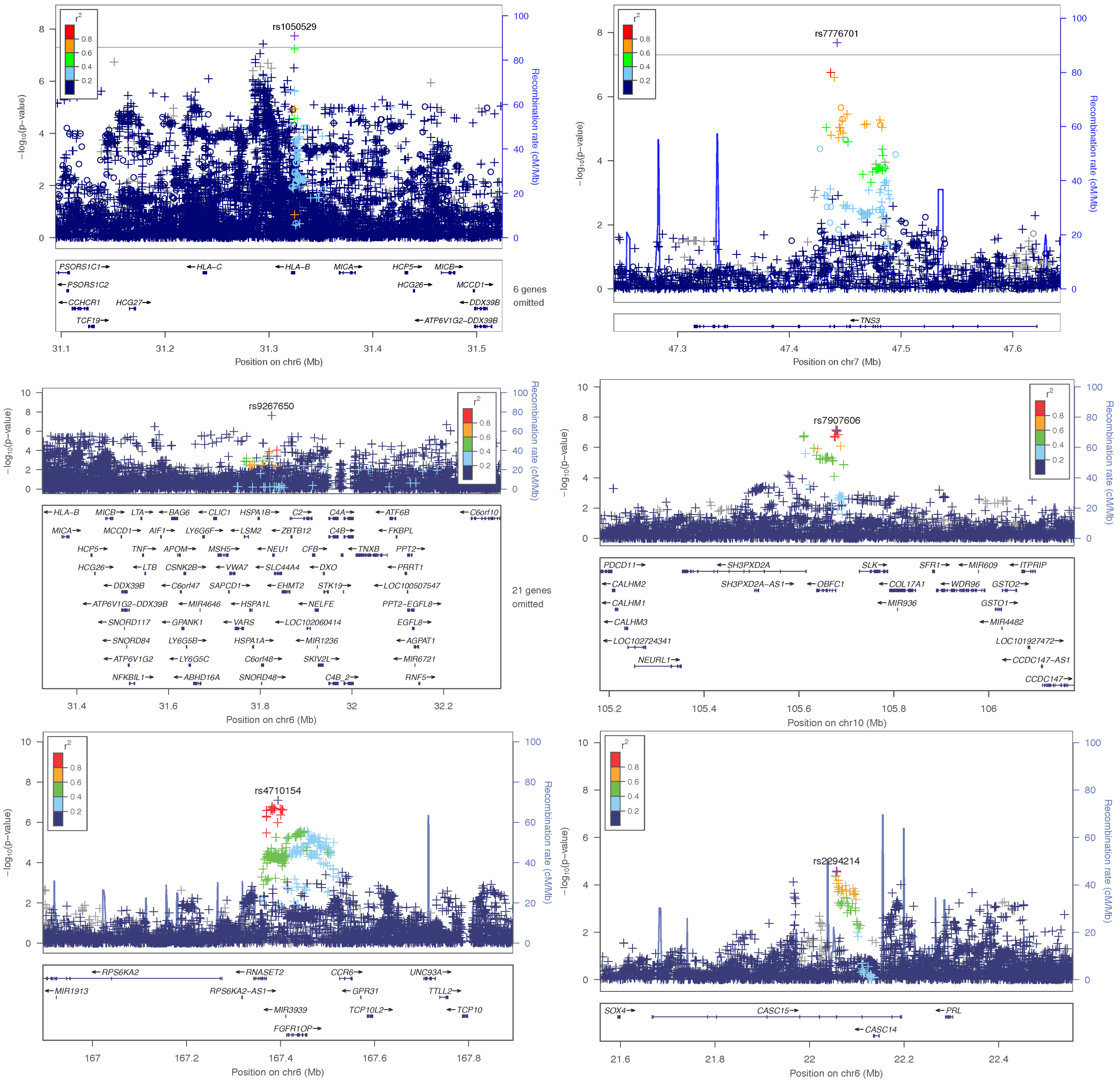

Supplementary Figure 12. Regional association plots for 6 of 14 novel, genome-wide significant BCC susceptibility loci are shown. Left to right, beginning from top left: 6p21.3 (rs1050529, *HLA-B*), 7p12.3 (rs7776701, *TNS3*), 6p21.3 (rs9267650, *NEU1*), 10q24.3 (rs7907606, *OBFC1*), 6q27 (rs4710154, *MIR3939*), 6p22.3 (rs2294214, *CASC15*). Each plot displays -log$_{10}$(*P* value) versus genomic position based on stage 1 logistic regression association testing.

Supplementary Table 1. Gender and age of BCC cases and controls from two-stage GWAS

	Status	n (%)	Male (%)	Age < 31 yr	Age 31-45	Age 46-60	Age > 60
**23andMe (Stage 1, n = 287197)**	*Cases*	12945 (4.5)	6700 (52)	42 (0.3)	650 (5)	3194 (25)	9059 (70)
	Controls	274252 (95.5)	148415 (54)	39673 (14)	83162 (30)	74977 (27)	76440 (28)
**Harvard (Stage 2, n = 17044)**							
Affy	*Cases*	1777 (24.7)	834 (46.9)	5 (0.3)	500 (28.1)	978 (55.0)	294 (16.5)
	Controls	5411 (75.3)	2385 (44.1)	43 (0.8)	2068 (38.2)	2533 (46.8)	767 (14.2)
Illumina	*Cases*	1268 (25.6)	368 (29.0)	6 (0.5)	472 (37.2)	678 (53.5)	112 (8.8)
	Controls	3685 (74.4)	1029 (27.9)	33 (0.9)	1681 (45.6)	1692 (45.9)	279 (7.6)
Omni	*Cases*	1197 (24.4)	447 (37.3)	4 (0.3)	403 (33.7)	645 (53.9)	145 (12.1)
	Controls	3706 (75.6)	1238 (33.4)	34 (0.9)	1671 (45.1)	1651 (44.5)	350 (9.4)
**All, Stage 2**	*Cases*	4242 (24.9)	1649 (38.9)	15 (0.4)	1375 (32.4)	2301 (54.2)	551 (13.0)
	Controls	12802 (75.1)	4652 (36.3)	110 (0.9)	5420 (42.3)	5876 (45.9)	1396 (10.9)
**Combined meta-analysis (n = 304241)**	*Cases*	17187 (5.6)	8349 (49)	57 (0.3)	2025 (12)	5495 (32)	9610 (56)
	Controls	287054 (94.4)	153067 (53)	39783 (14)	88582 (31)	80853 (28)	77836 (27)

Counts and percentages for cases and controls (n (%)) are listed above, stratified by stage of GWAS. We also report number and percentage of male subjects, subjects with age < 31 years, subjects with age 30-45 years, subjects with age 45-60 years, and subjects with age > 60 years. Stage 2 cases and controls are further subdivided based on platform used for genotyping.

Supplementary Table 2. Sensitivity and specificity of self-report data with respect to BCC diagnosis

n = 190	Disease (+)	Disease (-)
Self-report (+)	42	1
Self-report (-)	3	144
	**Sensitivity = 93%**	
	**Specificity = 99%**	

Within table, from left to right, are counts for true positives, false positives, false negatives, and true negatives. Data from 190 randomly selected patients at Stanford outpatient clinics.

Supplementary Table 3. Association of 28 imputed and genotyped index SNPs from stage 1 with BCC

Locus	Nearby genes	Imputed SNP	Imputed position	Imputation *P*-value	Genotyped SNP	Genotyped position	Genotyped *P*-value
6p25.3	*IRF4*	rs12203592	396321	7.9E-128	rs12210050	475489	1.6E-40
16q24.3	*MC1R*	rs1805007	89986117	3.5E-48	rs1805007	89986117	1.4E-45
20p13	*TGM3*	rs214785	2283457	4.4E-26	rs214796	2286343	3.8E-26
5p13.2	*SLC45A2*	rs35407	33946571	5.8E-26	rs16891982	33951693	3.2E-36
20q11.22	*RALY*	rs6059655	32665748	2.5E-22	rs910873	33171772	2.4E-13
1p36.13	*RCC2*	rs57142672	17744536	9.3E-21	rs6688886	17753639	1.9E-20
10p14	*GATA3*	rs73635312	8935998	8.5E-20	rs290903	8936140	8.5E-16
11q14.3	*TYR*	rs1126809	89017961	7.7E-19	rs1393350	89011046	1.1E-16
5p15.33	*CLPTM1L*	rs421284	1325590	2.0E-17	rs401681	1322087	3.4E-16
2q33.1	*ALS2CR12/CASP8*	rs2080303	202165206	1.4E-14	rs10931936	202143928	1.2E-13
1q42.13	*RHOU*	rs61824911	228979737	4.4E-14	rs71074	229003739	4.5E-13
3p13	*FOXP1/EIF4E3*	rs2116709	71621669	8.7E-14	rs9828619	71626123	1.4E-13
15q13.1	*OCA2/HERC2*	rs12916300	28410491	1.5E-13	rs16950987	28526228	6.0E-07
8q22.2	*RGS22*	rs141115006	101005020	2.4E-13	rs7006527	101024505	1.4E-11
9p22.2	*BNC2*	rs10810657	16884586	4.9E-13	rs2153271	16864521	5.3E-11
8q21.11	*ZFHX4*	rs10093547	77474545	7.4E-12	rs17431544	77467488	1.4E-11
3q28	*LPP*	rs191177147	188092546	2.2E-11	rs9851967	188087628	3.5E-10
6p21.32	*HLA-DQB1/DQA2*	rs9275642	32684935	7.7E-11	rs4947342	32653070	3.3E-09
9p21.3	*CDKN2B*	rs7874604	22054690	1.8E-10	rs7044859	22018781	3.1E-09
12q13.13	*KRT5*	rs11170164	52913668	2.7E-10	rs11170164	52913668	1.9E-10
7q22.1	*CUX1*	rs73183643	101418653	4.6E-10	rs11977362	101381523	3.3E-06
8q21.13	*ZBTB10*	rs11993814	81389791	1.4E-09	rs7830160	81381871	1.9E-09
21q22.3	*LINC00111*	rs2776353	43089017	2.3E-09	rs2776348	43086944	3.6E-08
13q34	*ATP11A*	rs1765871	113533651	4.9E-09	rs1046793	113539894	1.8E-08
19p13.3	*TICAM1/PLIN3*	rs10425559	4837487	1.5E-08	rs6510827	4830628	7.5E-08
6p21.33	*HLA-B*	rs1050529	31324615	1.8E-08	rs2524043	31257012	4.5E-05
7p12.3	*TNS3*	rs7776701	47442633	2.1E-08	rs834602	47446215	1.9E-06
22q12.1	*TTC28*	rs78097823	28648766	4.7E-08	rs9625433	28620996	4.5E-05

For each index SNP from stage 1, we show the best association test result for a nearby SNP that was genotyped on our V3 and/or V4 arrays. "Imputed *P*-value" refers to the association (via logistic regression) between the stage 1 imputed index SNPs and BCC. "Genotyped *P*-value" refers to the association (via logistic regression) between stage 1 genotyped SNPs and BCC.

Supplementary Table 4. Meta-analysis for 5 SNPs using subsets with high imputation quality in stage 2

				Stage 2							Meta-analysis				
SNP	Region	Gene	Maj/ min	*P*	*OR*	95% CI	Affy	Illum	Omni	Avg r^2	*P*	*OR*	95% CI	P_{het}	I^2
rs12203592	6p25.3	*IRF4*	C/T	3.1×10^{-10}	1.31	(1.21, 1.43)	-	1.00	1.00	1.00	1.5×10^{-151}	1.48	(1.44, 1.53)	0.00	88%
rs11170164	12q13.13	*KRT5*	C/T	3.4×10^{-5}	1.30	(1.15, 1.47)	-	0.85	0.99	0.92	1.2×10^{-14}	1.19	(1.14, 1.24)	0.13	56%
rs10425559	19p13.3	*PLIN3*	G/A	8.4×10^{-1}	0.99	(0.93, 1.06)	-	0.88	1.00	0.94	2.8×10^{-8}	0.93	(0.91, 0.96)	0.05	74%
rs9267650	6p21.33	*NEU1*	A/T	3.6×10^{-1}	1.08	(0.92, 1.27)	-	0.86	0.98	0.92	1.5×10^{-8}	1.17	(1.11, 1.24)	0.29	9%
rs12210050	6p25.3	*EXOC2*	C/T	3.3×10^{-5}	1.17	(1.09, 1.27)	1.00	1.00	-	1.00	1.0×10^{-49}	1.25	(1.21, 1.29)	0.07	69%

For each SNP, we report rsID, genetic locus, gene, major and minor alleles, *P* value for stage 2, odds ratio (for stage 2 overall, along with 95% CI), average imputation r^2 (Affy Illumina, Omni, stage 2 overall), *P* value for meta-analysis, odds ratio (for meta-analysis overall, plus 95% CI), and P_{het} and I^2 for meta-analysis. *P* values generated via logistic regression.

Supplementary Table 5. Heterogeneity analysis results between stage 2 genotyping and imputation data

				Imputation								Genotyping					
SNP	Region	Gene	Maj/ Min	MAF	*P*	OR	95% CI	Affy	Illum	Omni	Avg r^2	MAF	*P*	OR	95% CI	P_{het}	I^2
rs12916300	15q13.1	*HERC2*	T/C	0.35	5.0×10^{-4}	0.84	(0.76, 0.93)	0.47	0.31	0.11	0.30	0.21	3.0×10^{-1}	0.91	(0.75, 1.09)	0.47	0%
rs35407	5p13.2	*SLC45A2*	G/A	0.05	7.4×10^{-5}	0.71	(0.61, 0.84)	0.60	0.58	0.33	0.50	0.02	8.2×10^{-4}	0.43	(0.26, 0.71)	0.06	72%

For each SNP, we report rsID, genetic locus, gene, major and minor alleles, minor allele frequency (MAF), *P* value and odds ratio (for stage 2 imputation, along with 95% CI), average imputation r^2 (Affy, Illumina, Omni, stage 2 overall), MAF for genotyping, *P* value and odds ratio (for stage 2 genotyping, along with 95% CI), and P_{het} and I^2. *P* values generated via logistic regression.

Supplementary Table 6. Effect sizes of 28 index SNPs in stage 1 stratified by age

		Age interval				
**SNP**	**Gene**	(0,57]	(57,65]	(65,72]	(72,72+]	***P-value***
rs12203592	*IRF4*	0.509	0.395	0.434	0.275	1.30E-05
rs1805007	*MC1R*	0.406	0.345	0.392	0.200	0.0063
rs214785	*TGM3*	-0.246	-0.207	-0.118	-0.154	0.044
rs35407	*SLC45A2*	0.725	0.478	0.650	0.255	0.0065
rs6059655	*RALY*	-0.264	-0.284	-0.197	-0.187	0.061
rs57142672	*RCC2*	0.140	0.140	0.134	0.114	0.8
rs73635312	*GATA3*	0.257	0.223	0.111	0.145	0.038
rs1126809	*TYR*	-0.133	-0.175	-0.102	-0.114	0.18
rs421284	*CLPTM1L*	0.119	0.118	0.131	0.091	0.88
rs2080303	*ALS2CR12/CASP8*	0.130	0.129	0.118	0.098	0.99
rs61824911	*RHOU*	0.189	0.092	0.072	0.126	0.067
rs2116709	*FOXP1/EIF4E3*	-0.115	-0.105	-0.110	-0.114	0.99
rs12916300	*OCA2/HERC2*	0.185	0.127	0.114	0.106	0.25
rs141115006	*RGS22*	-0.127	-0.123	-0.132	-0.182	0.66
rs10810657	*BNC2*	-0.146	-0.078	-0.105	-0.082	0.24
rs10093547	*ZFHX4*	0.171	0.159	0.269	0.198	0.5
rs191177147	*LPP*	0.123	0.057	0.122	0.106	0.3
rs9275642	*HLA-DQB1/DQA2*	-0.125	-0.116	-0.097	-0.123	0.9
rs7874604	*CDKN2B*	0.078	0.076	0.111	0.115	0.78
rs11170164	*KRT5*	0.225	0.143	0.198	0.049	0.079
rs73183643	*CUX1*	0.141	0.128	0.061	0.084	0.42
rs11993814	*ZBTB10*	-0.060	-0.096	-0.104	-0.115	0.54
rs2776353	*LINC00111*	-0.103	-0.100	-0.097	-0.052	0.6
rs1765871	*ATP11A*	-0.074	-0.049	-0.123	-0.072	0.32
rs10425559	*TICAM1/PLIN3*	0.094	0.086	0.042	0.091	0.36
rs1050529	*HLA-B*	-0.124	-0.084	-0.068	-0.131	0.35
rs7776701	*TNS3*	-0.132	-0.086	-0.057	-0.033	0.048
rs78097823	*TTC28*	0.078	0.289	0.180	0.185	0.11

The table shows effect sizes (for each SNP reaching genome-wide significance in stage 1) computed in each age interval, as well as the *P* value for the test of genotype interaction with age interval. Stage 1 cohort was divided into four age intervals with similar effective sample sizes based on case and control sample counts. For all these association tests, the same covariates were used as in stage 1: age, sex, and five principal components. Thus, association tests within a specific age interval were still adjusted for age as a continuous covariate. Mean age for the 274,252 controls was 48.9 (SD 16.2); for the 12,945 cases, mean age was 65.5 (SD 11.5).

Supplementary Table 7. Gender-based logistic regression results for 28 index SNPs in stage 1

SNP	Gene	M_{effect}	M_{SE}	M_P	F_{effect}	F_{SE}	F_P	*P*
rs12203592	*IRF4*	0.455	0.022	7.0E-88	0.366	0.023	6.0E-55	0.040
rs1805007	*MC1R*	0.374	0.031	4.1E-32	0.316	0.031	1.8E-22	0.286
rs214785	*TGM3*	-0.167	0.023	1.2E-12	-0.199	0.023	4.3E-17	0.213
rs35407	*SLC45A2*	0.505	0.071	1.9E-14	0.541	0.074	1.9E-15	0.751
rs6059655	*RALY*	-0.279	0.032	1.3E-17	-0.194	0.032	4.4E-09	0.088
rs57142672	*RCC2*	0.143	0.019	1.6E-13	0.125	0.020	2.1E-10	0.612
rs73635312	*GATA3*	0.227	0.028	2.2E-16	0.141	0.028	3.3E-07	0.047
rs1126809	*TYR*	-0.128	0.020	2.8E-10	-0.140	0.021	1.5E-11	0.521
rs421284	*CLPTM1L*	0.116	0.019	4.7E-10	0.120	0.019	3.0E-10	0.783
rs2080303	*ALS2CR12/CASP8*	0.135	0.021	1.1E-10	0.105	0.021	8.2E-07	0.425
rs61824911	*RHOU*	0.139	0.021	9.1E-11	0.102	0.022	3.9E-06	0.300
rs2116709	*FOXP1/EIF4E3*	-0.133	0.020	2.6E-11	-0.087	0.020	1.7E-05	0.136
rs12916300	*OCA2/HERC2*	0.136	0.025	2.2E-08	0.133	0.025	9.3E-08	0.761
rs141115006	*RGS22*	-0.140	0.026	7.7E-08	-0.144	0.027	6.9E-08	0.819
rs10810657	*BNC2*	-0.104	0.019	4.8E-08	-0.102	0.020	2.0E-07	0.986
rs10093547	*ZFHX4*	0.217	0.041	7.8E-08	0.191	0.042	2.8E-06	0.755
rs191177147	*LPP*	0.098	0.021	3.4E-06	0.111	0.021	2.2E-07	0.517
rs9275642	*HLA-DQB1/DQA2*	-0.137	0.025	2.7E-08	-0.100	0.025	5.7E-05	0.346
rs7874604	*CDKN2B*	0.088	0.020	1.5E-05	0.106	0.021	3.7E-07	0.591
rs11170164	*KRT5*	0.186	0.033	3.4E-08	0.133	0.034	1.4E-04	0.303
rs73183643	*CUX1*	0.094	0.023	3.2E-05	0.115	0.023	5.1E-07	0.391
rs11993814	*ZBTB10*	-0.110	0.022	3.5E-07	-0.083	0.022	1.6E-04	0.413
rs2776353	*LINC00111*	-0.084	0.020	3.8E-05	-0.097	0.021	3.1E-06	0.623
rs1765871	*ATP11A*	-0.082	0.019	1.0E-05	-0.080	0.019	2.6E-05	0.981
rs10425559	*TICAM1/PLIN3*	0.093	0.019	1.1E-06	0.067	0.020	6.3E-04	0.458
rs1050529	*HLA-B*	-0.095	0.025	1.6E-04	-0.116	0.026	6.4E-06	0.486
rs7776701	*TNS3*	-0.074	0.019	7.2E-05	-0.081	0.019	2.2E-05	0.711
rs78097823	*TTC28*	0.212	0.045	4.9E-06	0.166	0.046	4.0E-04	0.466

The table shows results from logistic regression models (for each SNP reaching genome-wide significance in stage 1) fit separately in male and female subsets of the stage 1 cohort, and a *P*-value from a likelihood ratio test for adding a gender by genotype interaction to the full model. “M” stands for male and “F” for female. The subscripts “SE” and “P” stand for “standard error” and “P-value”, respectively. Of the 274,252 controls, 54% were male; of the 12,945 cases, 52% were male.

Supplementary Table 8. Replication of 16 previously confirmed and 5 previously reported BCC-associated loci

					Stage 1		Stage 2		Meta-analysis		Prior Studies		
SNP	Region	Gene	Maj/ min	MAF (avg imputation r^2)	*P*	*OR*	*P*	*OR*	*P*	*OR*	*P*	*OR*	Ref
rs12203592	6p25.3	*IRF4**	C/T	0.17 (0.99)	1.5×10^{-138}	1.51	1.5×10^{-11}	1.31	2.4×10^{-152}	1.48	1.6×10^{-3}	1.32	a
rs1805007	16q24.3	*MC1R*	C/T	0.07 (1.0)	4.0×10^{-52}	1.41	1.9×10^{-9}	1.34	2.5×10^{-63}	1.40	4.3×10^{-17}	1.55	4
rs12210050	6p25.3	*EXOC2*	C/T	0.17 (0.99)	3.3×10^{-45}	1.27	4.5×10^{-7}	1.19	1.0×10^{-51}	1.25	9.9×10^{-10}	1.24	4
rs214785	20p13	*TGM3*	T/C	0.18 (0.99)	3.7×10^{-28}	1.2	1.2×10^{-5}	1.15	7.9×10^{-33}	1.19	5.5×10^{-17}	1.29	6
rs35407	5p13.2	*SLC45A2**	G/A	0.04 (0.98)	5.0×10^{-28}	0.6	7.4×10^{-5}	0.71	5.2×10^{-27}	0.63	1.6×10^{-12}	0.51	b
rs6059655	20q11.22	*RALY**	G/A	0.07 (0.99)	4.2×10^{-24}	1.26	2.8×10^{-3}	1.15	2.5×10^{-26}	1.24	1.2×10^{-6}	1.35	c**
rs57142672	1p36.13	*RCC2*	A/G	0.34 (0.99)	2.2×10^{-22}	1.14	3.4×10^{-3}	1.08	1.0×10^{-23}	1.13	4.4×10^{-12}	1.28	2
rs73635312	10p14	*GATA3*	G/A	0.14 (1.0)	2.4×10^{-21}	0.83	2.3×10^{-4}	0.87	2.8×10^{-23}	0.84	2.4×10^{-16}	0.74	7
rs1126809	11q14.3	*TYR**	G/A	0.28 (0.99)	2.6×10^{-20}	1.14	1.3×10^{-1}	1.04	2.5×10^{-19}	1.12	6.1×10^{-4}	1.14	c
rs421284	5p15.33	*CLPTM1L*	T/C	0.44 (0.99)	9.1×10^{-19}	0.89	2.8×10^{-2}	0.94	1.1×10^{-18}	0.90	3.7×10^{-12}	0.80	3
rs2080303	2q33.1	*ALS2CR12*	C/T	0.32 (0.89)	1.1×10^{-15}	1.13	1.9×10^{-4}	1.11	7.4×10^{-19}	1.13	1.5×10^{-9}	1.15	7
rs61824911	1q42.13	*RHOU*	A/G	0.28 (0.90)	3.7×10^{-15}	1.13	1.4×10^{-1}	1.05	1.1×10^{-14}	1.11	5.9×10^{-12}	1.28	2
rs12916300	15q13.1	*OCA2**	T/C	0.29 (0.79)	1.4×10^{-14}	0.87	5.0×10^{-4}	0.84	8.2×10^{-17}	0.87	1×10^{-2}	0.70	a
rs141115006	8q22.2	*RGS22*	C/T	0.17 (0.95)	2.3×10^{-14}	0.87	5.1×10^{-3}	0.91	2.0×10^{-15}	0.88	8.7×10^{-13}	0.77	6
rs10093547	8q21.11	*ZFHX4*	T/G	0.06 (0.95)	9.6×10^{-13}	0.82	2.7×10^{-4}	0.82	4.6×10^{-15}	0.82	3.5×10^{-12}	0.70	7
rs7874604	9p21.3	*CDKN2B*	T/C	0.46 (0.84)	3.0×10^{-11}	0.91	3.8×10^{-3}	0.92	4.5×10^{-13}	0.91	3.0×10^{-10}	0.83	6
rs11170164	12q13.13	*KRT5*	C/T	0.08 (1.0)	4.7×10^{-11}	1.17	3.7×10^{-6}	1.26	1.1×10^{-15}	1.19	9.3×10^{-9}	1.29	7
rs78378222	17p13.1	*TP53*	T/G	0.01 (0.87)	3.5×10^{-8}	1.39	4.0×10^{-3}	1.54	1.8×10^{-10}	1.41	2.2×10^{-20}	2.16	5
rs157935	7q32.3	*KLF14*	T/G	0.29 (1.0)	6.5×10^{-5}	0.94	2.3×10^{-2}	0.94	4.5×10^{-6}	0.94	8.5×10^{-11}	0.81	6
rs57244888	2p24.3	*MYCN*	T/C	0.12 (0.92)	6.6×10^{-5}	0.92	4.4×10^{-1}	0.97	1.2×10^{-4}	0.93	4.7×10^{-12}	0.76	7
rs7335046	13q32.3	*UBAC2*	C/G	0.12 (0.99)	5.6×10^{-1}	1.01	2.5×10^{-4}	1.15	2.6×10^{-2}	1.04	2.9×10^{-8}	1.26	4

16 loci previously confirmed as associated with BCC via prior GWAS ($P < 5\times10^{-8}$) are listed, 12 of which independently reached genome-wide significance ($P < 5\times10^{-8}$) in this study. 5 additional BCC loci, previously reported via candidate gene studies, are indicated via asterisks; these loci are pigmentation-related, and all 5 reached genome-wide significance in this study. We report the most significant SNP at each locus based on stage 1 data. Additionally, we report major allele, minor allele, minor allele frequency (MAF) as calculated from stage 1 data, average imputation r^2 (a measure of imputation quality) for stage 1, and odds ratio (OR) with *P* value for each stage, calculated with respect to the minor allele via logistic regression. The right-most 3 columns list *P* value and OR from prior publications for each locus, along with corresponding reference number ("Ref") as listed in main text. Statistics for effect heterogeneity (P_{het} and I^2) are included in Supplementary Table 9.

a. Han, J. *et al.* A germline variant in the interferon regulatory factor 4 gene as a novel skin cancer risk locus. *Cancer Res.* 71, 1533 1539 (2011).
b. Stacey, S. N. *et al.* New common variants affecting susceptibility to basal cell carcinoma. *Nat. Genet.* 41, 909–914 (2009).
c. Gudbjartsson, D. F. *et al.* ASIP and TYR pigmentation variants associate with cutaneous melanoma and basal cell carcinoma. *Nat. Genet.* 40, 886–891 (2008).
**This publication reports *P* value and OR with respect to a two-SNP haplotype, rs1015362[G] and rs4911414[T], at the *RALY-ASIP* locus

Supplementary Table 9. Imputation and effect heterogeneity statistics for SNPs at previously reported loci

SNP	Gene	Maj/ min	Stage 1			Stage 2					Meta-analysis	
			MAF	avg r^2	min r^2	MAF	Affy r^2	Illum r^2	Omni r^2	Overall avg r^2	P_{het}	I^2
rs12203592	*IRF4**	C/T	0.17	0.99	0.98	0.17	0.32	1.00	1.00	0.77	0.00	91%
rs1805007	*MC1R*	C/T	0.07	1.00	1.00	0.07	1.00	0.82	0.88	0.90	0.32	0%
rs12210050	*EXOC2*	C/T	0.17	0.99	0.98	0.17	1.00	1.00	0.54	0.84	0.13	56%
rs214785	*TGM3*	T/C	0.18	0.99	0.98	0.18	1.00	0.99	0.99	0.99	0.27	17%
rs35407	*SLC45A2**	G/A	0.04	0.98	0.88	0.05	0.60	0.58	0.33	0.50	0.06	71%
rs6059655	*RALY**	G/A	0.07	0.99	0.98	0.08	0.97	0.98	0.99	0.98	0.07	70%
rs57142672	*RCC2*	A/G	0.34	0.99	0.98	0.35	0.98	0.98	0.98	0.98	0.06	71%
rs73635312	*GATA3*	G/A	0.14	1.00	1.00	0.14	0.97	1.00	0.99	0.98	0.27	16%
rs1126809	*TYR**	G/A	0.28	0.99	0.98	0.27	0.98	0.97	0.96	0.97	0.00	88%
rs421284	*CLPTM1L*	T/C	0.44	0.99	0.99	0.44	0.99	0.98	0.99	0.99	0.03	78%
rs2080303	*ALS2CR12*	C/T	0.32	0.89	0.88	0.32	0.88	0.88	0.88	0.88	0.71	0%
rs61824911	*RHOU*	A/G	0.28	0.90	0.89	0.27	0.89	0.88	0.88	0.88	0.03	80%
rs12916300	*OCA2**	T/C	0.29	0.79	0.75	0.35	0.47	0.31	0.11	0.30	0.40	0%
rs141115006	*RGS22*	C/T	0.17	0.95	0.92	0.17	0.98	0.92	0.96	0.96	0.27	17%
rs10093547	*ZFHX4*	T/G	0.06	0.95	0.93	0.07	0.93	0.93	0.94	0.93	0.95	0%
rs7874604	*CDKN2B*	T/C	0.46	0.84	0.82	0.45	0.84	0.81	0.82	0.82	0.63	0%
rs11170164	*KRT5*	C/T	0.08	1.00	0.99	0.08	0.73	0.85	0.99	0.86	0.19	42%
rs78378222	*TP53*	T/G	0.01	0.87	0.82	0.01	0.79	0.79	0.79	0.79	0.53	0%
rs157935	*KLF14*	T/G	0.29	1.00	1.00	0.30	0.97	1.00	1.00	0.99	0.85	0%
rs57244888	*MYCN*	T/C	0.12	0.92	0.90	0.12	0.99	0.88	0.91	0.93	0.23	30%
rs7335046	*UBAC2*	C/G	0.12	0.99	0.97	0.12	0.98	0.98	0.99	0.98	0.00	89%

These loci are all previously reported in prior BCC GWAS publications (16 loci) or candidate gene studies (5 loci, asterisks). We report genetic context, major and minor alleles, stage 1 minor allele frequency (MAF), stage 1 average imputation r^2 (avg r^2), stage 1 minimum imputation r^2, stage 2 MAF, stage 2 average imputation r^2 for each genotyping platform (Affy, Illumina, Omni) and overall, and *P* value (P_{het}) and I^2 for effect heterogeneity pertaining to meta-analysis of combined stage 1-stage 2 data.

Supplementary Table 10. Meta-analysis for rs12913832 and rs1800407

				Stage 1					Stage 2								Meta-analysis				
SNP	Region	Gene	Maj/ min	MAF	*P*	*OR*	95% CI	Avg r^2	MAF	*P*	*OR*	95% CI	Affy	Illum	Omni	Avg r^2	*P*	*OR*	95% CI	P_{het}	I^2
rs12913832	15q13.1	*OCA2/ HERC2*	G/A	0.28	1.0×10^{-11}	0.87	(0.84, 0.91)	1.00	0.26	6.2×10^{-4}	0.89	(0.83, 0.95)	0.99	1.00	NA	1.00	4.4×10^{-14}	0.88	(0.85, 0.91)	0.73	0%
rs1800407	15q13.1	*OCA2*	C/T	0.07	4.1×10^{-8}	1.14	(1.09, 1.20)	1.00	0.08	2.0×10^{-1}	1.12	(0.94, 1.34)	NA	NA	1.00	1.00	3.2×10^{-8}	1.14	(1.09, 1.19)	0.46	0%

OCA2/HERC2 rs12913832 is in modest linkage disequilibrium with rs12916300 (r^2=0.66, D'=0.93). 15q13.1 (rs1800407; *OCA2* R419Q) is a previously confirmed pigmentation-related locus. For each SNP, we report rsID, genetic locus, gene, major and minor alleles, stage 1 minor allele frequency (MAF), *P* value for stage 1, odds ratio (for stage 1 overall, plus 95% CI), average imputation r^2 (for stage 1), MAF for stage 2, *P* value for stage 2, odds ratio (for stage 2 overall, plus 95% CI), average imputation r^2 (for Affy, Illumina, Omni, stage 2 overall), *P* value for meta-analysis, odds ratio (for meta-analysis overall, plus 95% CI), and P_{het} and I^2 for meta-analysis. *P* values generated via logistic regression.

Supplementary Table 11. Meta-analysis for rs16891982

				Stage 1					Stage 2								Meta-analysis				
SNP	Region	Gene	Maj/ min	MAF	*P*	*OR*	95% CI	Avg r^2	MAF	*P*	*OR*	95% CI	Affy	Illum	Omni	Avg r^2	*P*	*OR*	95% CI	P_{het}	I^2
rs16891982	5p13.2	*SLC45A2*	G/C	0.05	1.7×10^{-25}	0.59	(0.53, 0.65)	1.00	0.07	1.2×10^{-4}	0.75	(0.64, 0.87)	0.56	0.58	0.32	0.49	5.7×10^{-24}	0.64	(0.58, 0.70)	0.01	84%

rs16891982 (Phe374Leu) in *SLC45A2* is non-synonymous exonic SNP. rs16891982 is in modest LD with rs35407 (r^2=0.33, D'=1). For each SNP, we report rsID, genetic locus, gene, major and minor alleles, stage 1 minor allele frequency (MAF), *P* value for stage 1, odds ratio (for stage 1 overall, plus 95% CI), average imputation r^2 (for stage 1), MAF for stage 2, *P* value for stage 2, odds ratio (for stage 2 overall, plus 95% CI), average imputation r^2 (for Affy, Illumina, Omni, stage 2 overall), *P* value for meta-analysis, odds ratio (for meta-analysis overall, plus 95% CI), and P_{het} and I^2 for meta-analysis. *P* values generated via logistic regression.

Supplementary Table 12. Mutually adjusted analysis for rs12203592 and rs12210050 in region 6p25.3 using subsets with high imputation quality in stage 2

					Independent analysis results[c]			Mutually adjusted analysis results[c]		
SNP	Region	Gene	Maj/ Min	r^2	*P*	OR	95% CI	*P*	OR	95% CI
rs12203592[a]	6p25.3	*IRF4*	C/T	0.997	4.3×10^{-4}	1.24	(1.10,1.40)	9.9×10^{-3}	1.21	(1.05,1.38)
rs12210050[b]	6p25.3	*EXOC2*	C/T	0.996	1.1×10^{-2}	1.17	(1.04,1.32)	3.7×10^{-1}	1.07	(0.93,1.22)

The second signal in the 6p25.3 region peaked at rs12210050, located near *EXOC2*; this SNP was previously reported to be associated with BCC risk and replicated in our study. This SNP is not in strong linkage disequilibrium with the top SNP in 6p25.3, rs12203592 (r^2=0.26, D'= 0.51). We performed a conditional analysis mutually adjusting for both SNPs in stage 2, using a high-quality Illumina dataset (R^2 > 0.99 for both SNPs). The results demonstrate that rs12203592 remained significant but rs12210050 did not. For each SNP, we report rsID, genetic locus, gene, major and minor alleles, stage 2 average imputation r^2, and *P* value and OR (along with 95% CI) for independent analysis results and mutually adjusted analysis results, generated via logistic regression.

a. Mutually adjusted analysis adjusted by SNP rs12210050
b. Mutually adjusted analysis adjusted by top SNP rs12203592
c. Analysis was generated using subsets with high imputation quality in stage 2

Supplementary Table 13. Imputation and effect heterogeneity statistics for 14 novel genome-wide significant SNPs

			Stage 1			Stage 2					Meta-analysis	
SNP	Gene	Maj/ min	MAF	avg r^2	min r^2	MAF	Affy r^2	Illum r^2	Omni r^2	Overall avg r^2	P_{het}	I^2 (%)
rs2116709	*FOXP1*	A/T	0.40	0.91	0.90	0.41	0.90	0.89	0.90	0.89	0.58	0%
rs10810657	*BNC2*	A/T	0.41	0.98	0.97	0.40	0.98	0.97	0.97	0.97	0.89	0%
rs191177147	*LPP*	G/T	0.39	0.80	0.79	0.39	0.75	0.78	0.78	0.77	0.81	0%
rs9275642	*HLA-DQA2*	C/T	0.21	0.89	0.81	0.22[a]	a	a	a	a	1.00	0%
rs73183643	*CUX1*	G/A	0.24	0.96	0.93	0.24	0.98	0.94	0.92	0.94	0.76	0%
rs11993814	*ZBTB10*	C/T	0.26	1.00	0.98	0.25	0.98	0.99	1.00	0.99	0.26	21%
rs2776353	*LINC00111*	A/T	0.32	0.96	0.93	0.32	0.91	0.91	0.96	0.93	0.85	0%
rs10425559	*PLIN3*	G/A	0.40	0.97	0.89	0.40	0.28	0.88	1.00	0.72	0.05	74%
rs1050529	*HLA-B*	C/T	0.25	0.71	0.69	0.19[b]	b	b	b	b	0.91	0%
rs7776701	*TNS3*	C/T	0.48	0.98	0.97	0.48	0.98	0.98	0.98	0.98	0.04	77%
rs9267650	*NEU1*	A/T	0.05	0.98	0.98	0.05	0.66	0.86	0.98	0.83	0.26	23%
rs7907606	*OBFC1*	T/G	0.17	0.96	0.92	0.17	0.97	0.92	0.97	0.96	0.63	0%
rs4710154	*MIR3939*	A/T	0.32	0.93	0.93	0.31	0.91	0.92	0.92	0.91	0.53	0%
rs2294214	*CASC15*	A/C	0.32	0.95	0.93	0.30	0.82	0.95	0.74	0.84	0.07	69%

SNPs that met genome-wide significance ($P < 5 \times 10^{-8}$, via logistic regression) in stage 1 and/or overall meta-analysis are listed. Additionally, we report genetic context, major and minor alleles, stage 1 minor allele frequency (MAF), stage 1 average imputation r^2 (avg r^2), stage 1 minimum imputation r^2, stage 2 MAF, stage 2 average imputation r^2 for each genotyping platform (Affy, Illumina, Omni) and overall, and P value (P_{het}) and I^2 for effect heterogeneity pertaining to meta-analysis of combined stage 1-stage 2 data.

a. Due to low imputation quality in stage 2 (r^2 <0.3), genotyping results are utilized for this result.
b. Due to low imputation quality in stage 2 (r^2 <0.3), genotyping results are utilized for this result. rs1050529 is located at HLA region, harboring high genomic diversity. rs9266772 is used as proxy SNP for rs1050529 (r^2=0.569,D'=0.771).

Supplementary Table 14. Adjustment analysis for pigmentation phenotype in stage 2.

SNP	Region	Gene	Maj/ min	β	SE	*P* value	Adj β[a]	Adj SE[a]	Adj *P* value[a]	Note
rs12203592	6p25.3	*IRF4*	C/T	0.269	0.04	1.5E-11	0.281	0.045	3.1E-10	PC
rs1805007	16q24.3	*MC1R*	C/T	0.291	0.049	1.9E-09	0.223	0.054	3.0E-05	PC
rs12210050	6p25.3	*EXOC2*	C/T	0.177	0.035	4.5E-07	0.158	0.038	3.7E-05	PC
rs214785	20p13	*TGM3*	T/C	-0.143	0.033	1.2E-05	0.131	0.035	2.1E-04	PC
rs35407	5p13.2	*SLC45A2*	G/A	-0.336	0.085	7.4E-05	-0.189	0.09	3.5E-02	PC
rs6059655	20q11.22	*RALY*	G/A	0.14	0.047	2.8E-03	0.035	0.051	5.0E-01	PC
rs57142672	1p36.13	*RCC2*	A/G	-0.078	0.027	3.4E-03	0.1	0.029	4.8E-04	PC
rs73635312	10p14	*GATA3*	G/A	-0.139	0.038	2.3E-04	-0.158	0.041	1.1E-04	PC
rs1126809	11q14.3	*TYR*	G/A	0.043	0.029	1.3E-01	0.018	0.031	5.5E-01	PC
rs421284	5p15.33	*CLPTM1L*	T/C	0.057	0.026	2.8E-02	-0.072	0.028	1.0E-02	PC
rs2080303	2q33.1	*ALS2CR12*	C/T	0.108	0.029	1.9E-04	0.123	0.031	8.3E-05	PC
rs61824911	1q42.13	*RHOU*	A/G	-0.045	0.031	1.4E-01	0.056	0.033	9.4E-02	PC
rs12916300	15q13.1	*OCA2*	T/C	0.18	0.052	5.0E-04	-0.102	0.054	5.6E-02	PC
rs141115006	8q22.2	*RGS22*	C/T	-0.098	0.035	5.1E-03	-0.079	0.038	3.7E-02	PC
rs10093547	8q21.11	*ZFHX4*	T/G	0.2	0.055	2.7E-04	-0.254	0.06	2.3E-05	PC
rs7874604	9p21.3	*CDKN2B*	T/C	0.081	0.028	3.8E-03	-0.053	0.03	8.1E-02	PC
rs11170164	12q13.13	*KRT5*	C/T	0.232	0.05	3.7E-06	0.224	0.054	3.7E-05	PC
rs78378222	17p13.1	*TP53*	T/G	-0.433	0.15	4.0E-03	0.36	0.14	1.0E-02	PC
rs157935	7q32.3	*KLF14*	T/G	0.064	0.028	2.3E-02	-0.066	0.031	3.0E-02	PC
rs57244888	2p24.3	*MYCN*	T/C	0.031	0.041	4.4E-01	-0.009	0.044	8.4E-01	PC
rs7335046	13q32.3	*UBAC2*	C/G	-0.139	0.038	2.5E-04	0.134	0.041	9.5E-04	PC
rs2116709	3p13	*FOXP1*	A/T	0.094	0.027	6.1E-04	-0.094	0.03	1.4E-03	N
rs10810657	9p22.2	*BNC2*	A/T	0.107	0.027	5.7E-05	-0.085	0.029	3.0E-03	N
rs191177147	3q28	*LPP*	G/T	0.097	0.03	1.0E-03	0.083	0.032	9.1E-03	N
rs9275642	6p21.32	*HLA-DQA2*	C/T	-0.212	0.096	2.7E-02	-2.812	1.406	4.6E-02	N
rs73183643	7q22.1	*CUX1*	G/A	-0.115	0.031	2.3E-04	-0.132	0.034	8.8E-05	N
rs11993814	8q21.13	*ZBTB10*	C/T	-0.06	0.03	4.5E-02	-0.083	0.032	9.5E-03	N
rs2776353	21q22.3	*LINC00111*	A/T	0.096	0.029	7.7E-04	-0.106	0.031	5.5E-04	N
rs10425559	19p13.3	*PLIN3*	G/A	-0.007	0.035	8.4E-01	0.004	0.035	9.0E-01	N
rs1050529	6p21.33	*HLA-B*	C/T	-0.118	0.107	2.7E-01	-0.312	0.179	8.2E-02	N
rs7776701	7p12.3	*TNS3*	C/T	-0.017	0.026	5.1E-01	-0.005	0.028	8.7E-01	N
rs9267650	6p21.3	*NEU1*	A/T	-0.086	0.066	2.0E-01	0.076	0.072	2.9E-01	N
rs7907606	10q24.3	*OBFC1*	T/G	-0.077	0.034	2.4E-02	0.073	0.037	4.9E-02	N
rs4710154	6q27	*MIR3939*	A/T	-0.058	0.028	4.3E-02	0.068	0.031	2.7E-02	N
rs2294214	6p22.3	*CASC15*	A/C	-0.12	0.03	5.9E-05	0.135	0.032	2.9E-05	N

[a] Analysis was adjusted by hair color, tanning ability during adolescence, and number of blistering sunburns in the logistic regression association model. "β" refers to effect size, "SE" to standard error, "Adj" to adjusted, "PC" to previously confirmed susceptibility locus, and "N" to novel susceptibility locus.

Supplementary Table 15. **Interaction analysis for hair color, number of sunburns, tanning ability, and UVB exposure in stage 2.**

SNP	Region	Gene	Maj/ min	Stage 2					Note
				P for SNP	Int *P* hair color	Int *P* sunburn	Int *P* tan	Int *P* UVB	
rs12203592	6p25.3	*IRF4*	C/T	1.5E-11	0.0147	0.1269	0.0231	0.1574	PC
rs1805007	16q24.3	*MC1R*	C/T	1.9E-09	0.2617	0.0553	0.4527	0.8077	PC
rs12210050	6p25.3	*EXOC2*	C/T	4.5E-07	0.34	0.9371	0.2096	0.5712	PC
rs214785	20p13	*TGM3*	T/C	1.2E-05	0.6537	0.1576	0.7358	0.4297	PC
rs35407	5p13.2	*SLC45A2*	G/A	7.4E-05	0.7456	0.7548	0.9102	0.0587	PC
rs6059655	20q11.22	*RALY*	G/A	2.8E-03	0.0894	0.5644	0.5252	0.1697	PC
rs57142672	1p36.13	*RCC2*	A/G	3.4E-03	0.9476	0.6069	0.3238	0.5949	PC
rs73635312	10p14	*GATA3*	G/A	2.3E-04	0.5108	0.6284	0.8583	0.3658	PC
rs1126809	11q14.3	*TYR*	G/A	1.3E-01	0.5733	0.1951	0.0711	0.06	PC
rs421284	5p15.33	*CLPTM1L*	T/C	2.8E-02	0.1991	0.2146	0.4098	0.8742	PC
rs2080303	2q33.1	*ALS2CR12*	C/T	1.9E-04	0.9261	0.7925	0.0471	0.912	PC
rs61824911	1q42.13	*RHOU*	A/G	1.4E-01	0.5005	0.3275	0.8657	0.4982	PC
rs12916300	15q13.1	*OCA2*	T/C	5.0E-04	0.1479	0.1949	0.148	0.4302	PC
rs141115006	8q22.2	*RGS22*	C/T	5.1E-03	0.9034	0.6912	0.074	0.8917	PC
rs10093547	8q21.11	*ZFHX4*	T/G	2.7E-04	0.0971	0.811	0.859	0.4303	PC
rs7874604	9p21.3	*CDKN2B*	T/C	3.8E-03	0.1196	0.0281	0.4439	0.2258	PC
rs11170164	12q13.13	*KRT5*	C/T	3.7E-06	0.9566	0.1993	0.9099	0.6325	PC
rs78378222	17p13.1	*TP53*	T/G	4.0E-03	0.8911	0.7125	0.0881	0.1847	PC
rs157935	7q32.3	*KLF14*	T/G	2.3E-02	0.4197	0.7012	0.6323	0.5127	PC
rs57244888	2p24.3	*MYCN*	T/C	4.4E-01	0.8525	0.9742	0.5191	0.8522	PC
rs7335046	13q32.3	*UBAC2*	C/G	2.5E-04	0.4801	0.7122	0.8656	0.1632	PC
rs2116709	3p13	*FOXP1*	A/T	6.1E-04	0.017	0.8123	0.0234	0.0453	N
rs10810657	9p22.2	*BNC2*	A/T	5.7E-05	0.0268	0.6183	0.1503	0.4562	N
**rs191177147**	**3q28**	***LPP***	**G/T**	**1.0E-03**	**0.0002**	**0.9832**	**0.7416**	**0.8174**	**N**
rs9275642	6p21.32	*HLA-DQA2*	C/T	2.7E-02	0.8354	0.4512	0.2499	0.9685	N
rs73183643	7q22.1	*CUX1*	G/A	2.3E-04	0.7846	0.9846	0.7022	0.6498	N
rs11993814	8q21.13	*ZBTB10*	C/T	4.5E-02	0.4267	0.4773	0.6854	0.7465	N
rs2776353	21q22.3	*LINC00111*	A/T	7.7E-04	0.6476	0.7809	0.1028	0.2998	N
rs10425559	19p13.3	*PLIN3*	G/A	8.4E-01	0.0058	0.9691	0.9614	0.5868	N
rs1050529	6p21.33	*HLA-B*	C/T	2.7E-01	0.2365	0.162	0.7724	0.1615	N
rs7776701	7p12.3	*TNS3*	C/T	5.1E-01	0.6746	0.4589	0.3433	0.0521	N
rs9267650	6p21.3	*NEU1*	A/T	2.0E-01	0.7232	0.4631	0.688	0.7657	N
rs7907606	10q24.3	*OBFC1*	T/G	2.4E-02	0.0505	0.5912	0.0194	0.397	N
rs4710154	6q27	*MIR3939*	A/T	4.3E-02	0.0021	0.9838	0.8908	0.5559	N
rs2294214	6p22.3	*CASC15*	A/C	5.9E-05	0.2909	0.3244	0.3393	0.0895	N

"*P* for SNP" refers to logistic regression P-value in stage 2, "Int" to interaction, "PC" to previously confirmed susceptibility locus, and "N" to novel susceptibility locus.

Supplementary Table 16. Meta-analysis for 4 SNPs within *OBFC1,* in linkage disequilibrium with index SNP rs7907606

				Stage 1					Stage 2								Meta-analysis				
SNP	Region	Gene	Maj/ min	MAF	*P*	*OR*	95% CI	Avg r^2	MAF	*P*	*OR*	95% CI	Affy	Illum	Omni	Avg r^2	*P*	*OR*	95% CI	P_{het}	I^2
rs9420907	10q24.33	*OBFC1*	A/C	0.14	2.0×10^{-7}	1.10	(1.06, 1.14)	1.00	0.14	1.7×10^{-1}	1.05	(0.98, 1.13)	1.00	1.00	1.00	1.00	1.2×10^{-7}	1.09	(1.06, 1.13)	0.24	29%
rs9419958	10q24.33	*OBFC1*	C/T	0.14	1.9×10^{-7}	1.10	(1.06, 1.14)	1.00	0.14	1.7×10^{-1}	1.05	(0.98, 1.13)	1.00	1.00	1.00	1.00	1.1×10^{-7}	1.09	(1.06, 1.13)	0.23	30%
rs4387287	10q24.33	*OBFC1*	C/A	0.17	6.8×10^{-8}	1.10	(1.06, 1.14)	0.98	0.17	3.6×10^{-2}	1.07	(1, 1.15)	0.98	0.94	1.00	0.97	6.7×10^{-9}	1.09	(1.06, 1.13)	0.52	0%
rs10786775	10q24.33	*OBFC1*	C/G	0.10	4.4×10^{-6}	1.11	(1.06, 1.15)	1.00	0.10	6.9×10^{-2}	1.08	(0.99, 1.17)	0.99	1.00	1.00	1.00	7.0×10^{-7}	1.10	(1.06, 1.14)	0.62	0%

For each SNP, we report rsID, genetic locus, gene, major and minor alleles, stage 1 minor allele frequency (MAF), *P* value for stage 1, odds ratio (for stage 1 overall, plus 95% CI), average imputation r^2 (for stage 1), MAF for stage 2, *P* value for stage 2, odds ratio (for stage 2 overall, plus 95% CI), average imputation r^2 (for Affy, Illumina, Omni, stage 2 overall), *P* value for meta-analysis, odds ratio (for meta-analysis overall, plus 95% CI), and P_{het} and I^2 for meta-analysis. *P* values generated via logistic regression.

Supplementary Table 17. Meta-analysis for 2 SNPs within *LPP*, in linkage disequilibrium with index SNP rs191177147

				Stage 1					Stage 2								Meta-analysis				
SNP	Region	Gene	Maj/ min	MAF	*P*	*OR*	95% CI	Avg r^2	MAF	*P*	*OR*	95% CI	Affy	Illum	Omni	Avg r^2	*P*	*OR*	95% CI	P_{het}	I^2
rs1464510	3q28	*LPP*	C/A	0.44	7.3×10^{-11}	0.92	(0.89, 0.94)	1.00	0.45	1.8×10^{-3}	0.92	(0.88, 0.97)	0.98	0.97	0.99	0.98	5.5×10^{-13}	0.92	(0.9, 0.94)	0.83	0%
rs9860547	3q28	*LPP*	G/A	0.46	5.3×10^{-10}	1.09	(1.06, 1.12)	0.97	0.46	2.8×10^{-3}	1.08	(1.03, 1.14)	0.92	0.96	0.97	0.95	5.5×10^{-12}	1.09	(1.06, 1.11)	0.85	0%

For each *LPP* SNP, we report rsID, genetic locus, gene, major and minor alleles, stage 1 minor allele frequency (MAF), *P* value for stage 1, odds ratio (for stage 1 overall, plus 95% CI), average imputation r^2 (for stage 1), MAF for stage 2, *P* value for stage 2, odds ratio (for stage 2 overall, plus 95% CI), average imputation r^2 (for Affy, Illumina, Omni, stage 2 overall), *P* value for meta-analysis, odds ratio (for meta-analysis overall, plus 95% CI), and P_{het} and I^2 for meta-analysis. *P* values generated via logistic regression.

Supplementary Table 18. Meta-analysis for 1 SNP within *TICAM1,* in linkage disequilibrium with index SNP rs10425559

				Stage 1					Stage 2								Meta-analysis				
SNP	Region	Gene	Maj/ min	MAF	*P*	*OR*	95% CI	Avg r^2	MAF	*P*	*OR*	95% CI	Affy	Illum	Omni	Avg r^2	*P*	*OR*	95% CI	P_{het}	I^2
rs7255265	19p13.3	*TICAM1*	C/T	0.37	3.7×10^{-7}	0.93	(0.91, 0.96)	0.99	0.35	8.6×10^{-1}	0.99	(0.94, 1.06)	0.37	0.97	1.00	0.78	2.5×10^{-6}	0.94	(0.92, 0.97)	0.06	72%

For each SNP, we report rsID, genetic locus, gene, major and minor alleles, stage 1 minor allele frequency (MAF), *P* value for stage 1, odds ratio (for stage 1 overall, plus 95% CI), average imputation r^2 (for stage 1), MAF for stage 2, *P* value for stage 2, odds ratio (for stage 2 overall, plus 95% CI), average imputation r^2 (for Affy, Illumina, Omni, stage 2 overall), *P* value for meta-analysis, odds ratio (for meta-analysis overall, plus 95% CI), and P_{het} and I^2 for meta-analysis. *P* values generated via logistic regression.

Supplementary Table 19. Meta-analysis for 1 SNP in linkage disequilibrium with index SNP rs4710154

				Stage 1					Stage 2								Meta-analysis				
SNP	Region	Gene	Maj/ min	MAF	*P*	*OR*	95% CI	Avg r^2	MAF	*P*	*OR*	95% CI	Affy	Illum	Omni	Avg r^2	*P*	*OR*	95% CI	P_{het}	I^2
rs9355610	6q27	*RNASET2*	G/A	0.34	2.6×10^{-7}	1.07	(1.05, 1.1)	1.00	0.34	4.6×10^{-2}	1.05	(1, 1.11)	1.00	0.98	1.00	0.99	3.7×10^{-8}	1.07	(1.04, 1.1)	0.56	0%

For each SNP, we report rsID, genetic locus, gene, major and minor alleles, stage 1 minor allele frequency (MAF), *P* value for stage 1, odds ratio (for stage 1 overall, plus 95% CI), average imputation r^2 (for stage 1), MAF for stage 2, *P* value for stage 2, odds ratio (for stage 2 overall, plus 95% CI), average imputation r^2 (for Affy, Illumina, Omni, stage 2 overall), *P* value for meta-analysis, odds ratio (for meta-analysis overall, plus 95% CI), and P_{het} and I^2 for meta-analysis. *P* values generated via logistic regression.

Supplementary Table 20. Meta-analysis for 4 SNPs within *BNC2,* in linkage disequilibrium with index SNP rs10810657

				Stage 1					Stage 2								Meta-analysis				
SNP	Region	Gene	Maj/ min	MAF	*P*	*OR*	95% CI	Avg r^2	MAF	*P*	*OR*	95% CI	Affy	Illum	Omni	Avg r^2	*P*	*OR*	95% CI	P_{het}	I^2
rs62543565	9p22.2	*BNC2*	A/C	0.41	1.1×10^{-13}	0.90	(0.87, 0.93)	0.90	0.40	3.5×10^{-4}	0.91	(0.86, 0.96)	0.89	0.87	0.88	0.88	2.2×10^{-16}	0.90	(0.88, 0.92)	0.84	0.0%
rs10756819	9p22.2	*BNC2*	A/G	0.34	1.5×10^{-5}	0.94	(0.92, 0.97)	1.00	0.34	7.7×10^{-3}	0.93	(0.88, 0.98)	0.89	1.00	1.00	0.96	4.2×10^{-7}	0.94	(0.92, 0.96)	0.67	0.0%
rs2153271	9p22.2	*BNC2*	T/C	0.42	1.5×10^{-11}	0.91	(0.89, 0.94)	1.00	0.42	4.8×10^{-5}	0.90	(0.85, 0.95)	0.98	1.00	1.00	0.99	4.3×10^{-15}	0.91	(0.89, 0.93)	0.60	0%
rs12350739	9p22.2	*BNC2*	A/G	0.44	2.3×10^{-12}	0.91	(0.89, 0.93)	0.97	0.43	7.2×10^{-6}	0.89	(0.84, 0.93)	0.90	0.97	0.96	0.94	1.4×10^{-16}	0.90	(0.88, 0.93)	0.41	0.0%

For each *BNC2* SNP, we report rsID, genetic locus, gene, major and minor alleles, stage 1 minor allele frequency (MAF), *P* value for stage 1, odds ratio (for stage 1 overall, plus 95% CI), average imputation r^2 (for stage 1), MAF for stage 2, *P* value for stage 2, odds ratio (for stage 2 overall, plus 95% CI), average imputation r^2 (for Affy, Illumina, Omni, stage 2 overall), *P* value for meta-analysis, odds ratio (for meta-analysis overall, plus 95% CI), and P_{het} and I^2 for meta-analysis. *P* values generated via logistic regression.
