# Supplementary material for: Genome-wide association study identifies 14 novel risk alleles associated with basal cell carcinoma
Source: Nat Commun. 2016 Aug 19;7:12510. doi: 10.1038/ncomms12510 (PMC4992160; doi:10.1038/ncomms12510)
Supplement: Peer review file [file ncomms12510-s2.pdf]

Transferred manuscripts:

Editorial Note: this manuscript has been previously reviewed at another journal that is not operating a transparent peer review scheme. This document only contains reviewer comments and rebuttal letters for versions considered at Nature Communications.

Reviewer #1 (Remarks to the Author)

I am satisfied that the authors have adequately addressed the points from Reviewer 2 - answering the questions on heritability, SNP environment interaction and EQTL analysis .

Reviewer #3 (Remarks to the Author)

The authors have extensively addressed all my comments and were very responsive to all concerns and suggestions. In addition they have performed numerous additional important analyses as recommended resulting in much clearer and stronger manuscript. I have no further comments.
